# Supplementary material for: The determination of thiocyanate in the blood plasma and holding water of Amphiprion clarkii after exposure to cyanide
Source: PeerJ. 2021 Dec 7;9:e12409. doi: 10.7717/peerj.12409 (PMC8663612; doi:10.7717/peerj.12409)
Supplement: Supplemental Information 15 [file peerj-09-12409-s015.pdf]

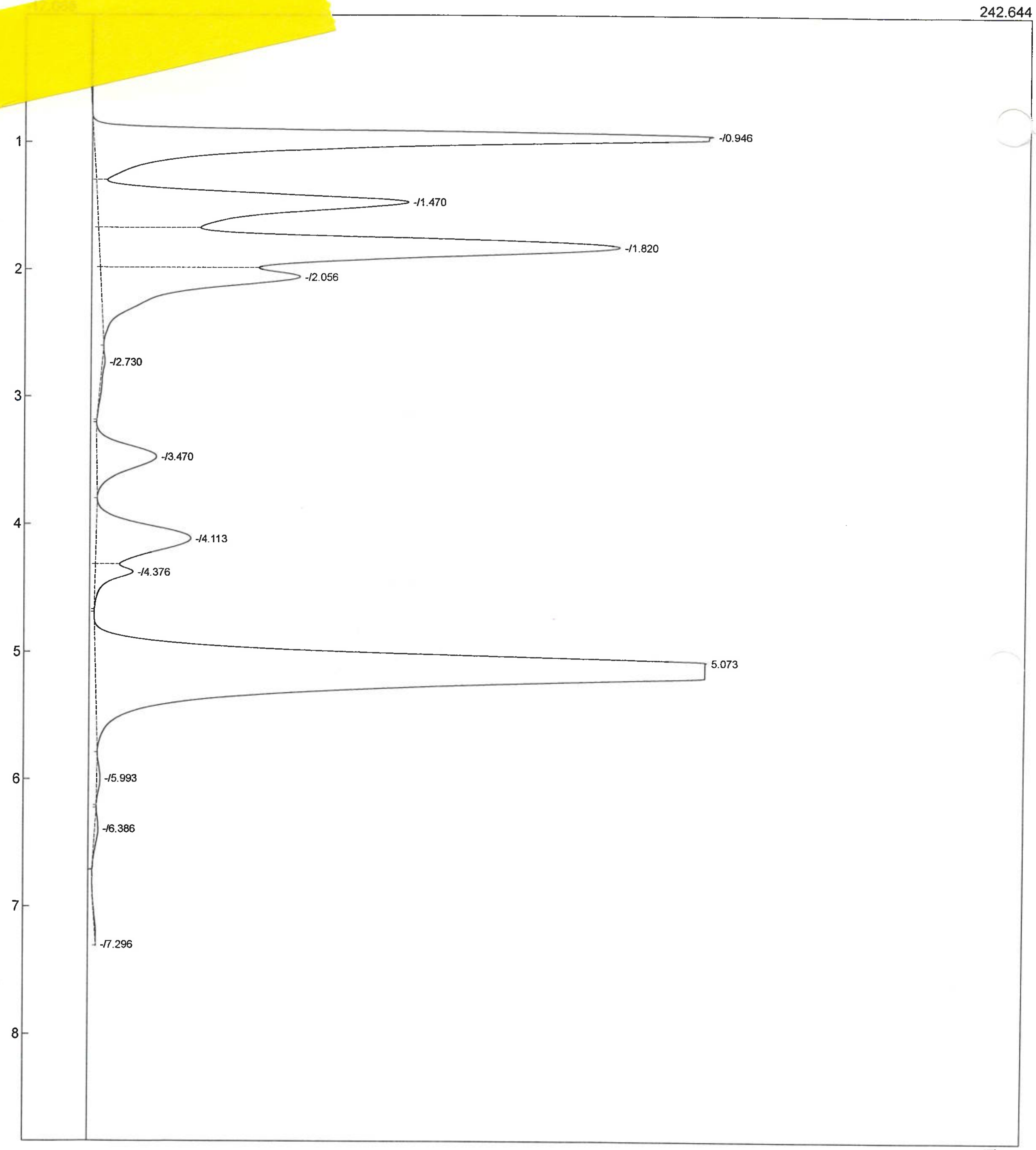

Component	Retention	Area	External	Units
	5.073	2934.9791	0.0000	
		2934.9791	0.0000	

F14 top

7/18/18

FJ 7 157

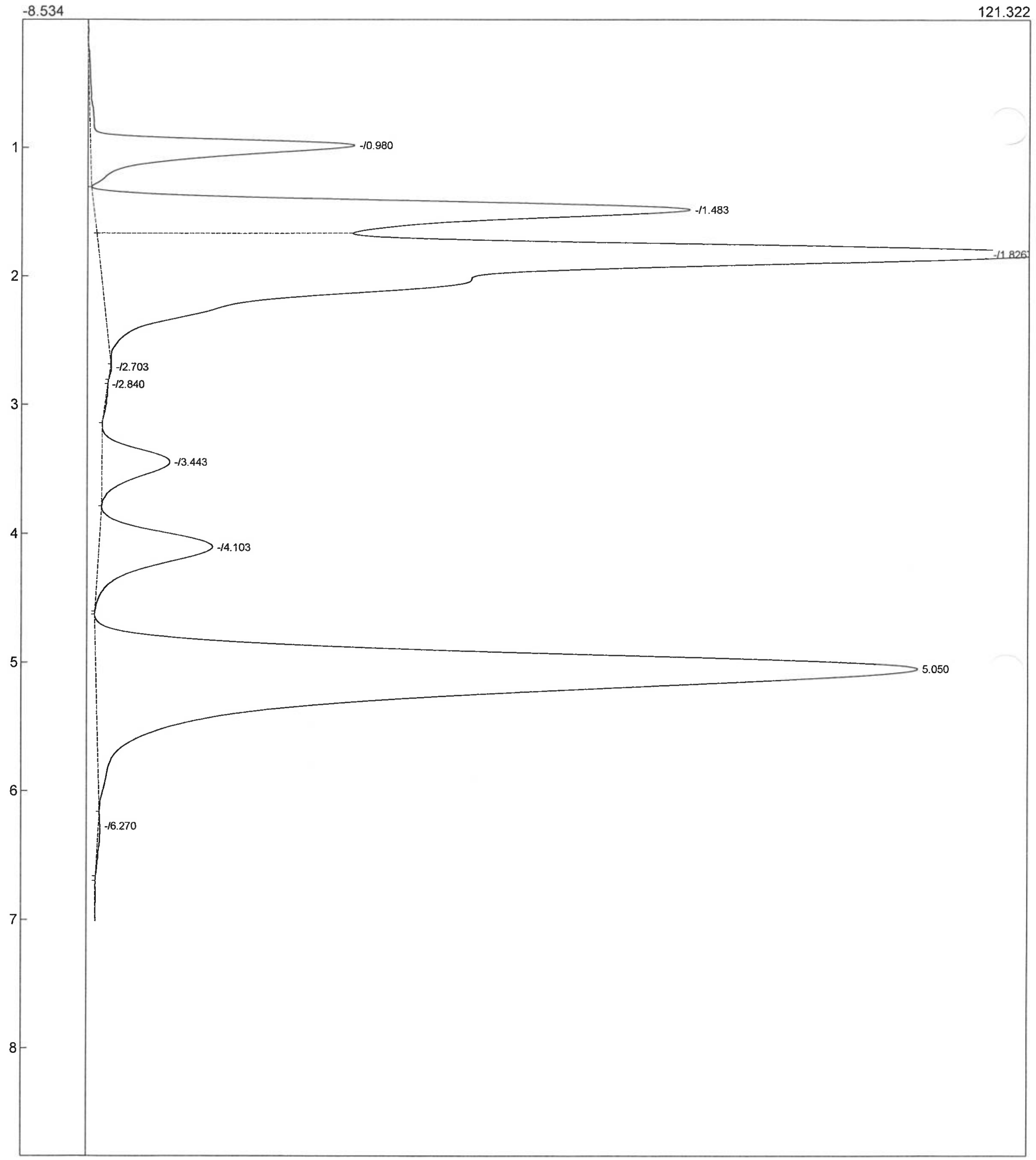

Component	Retention	Area	External	Units
	5.050	2320.5296	0.0000	
		2320.5296	0.0000	

8/18/18

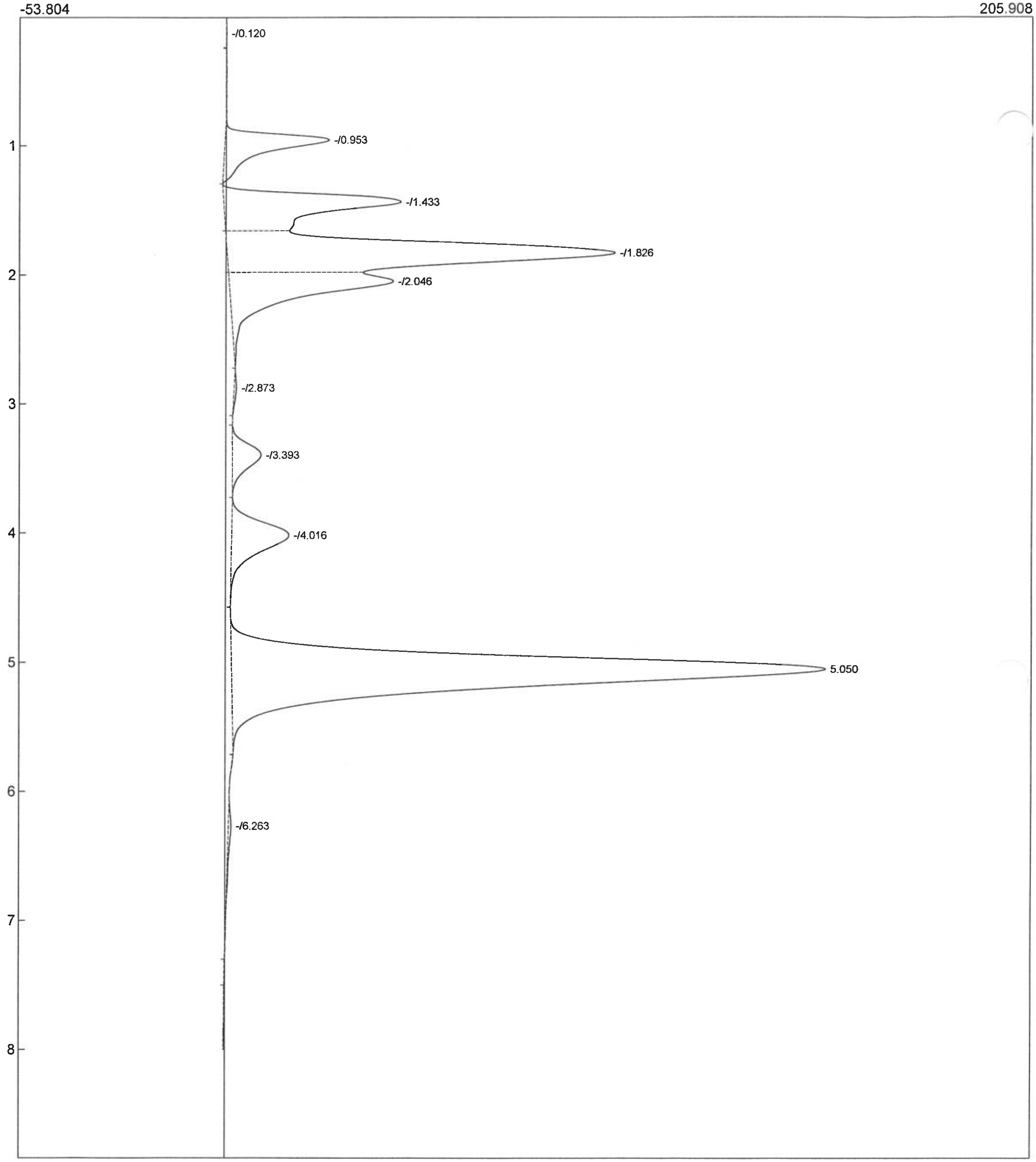

Component	Retention	Area	External	Units
	5.050	2446.2914	0.0000	
		2446.2914	0.0000	

Fish 159
7/18/18

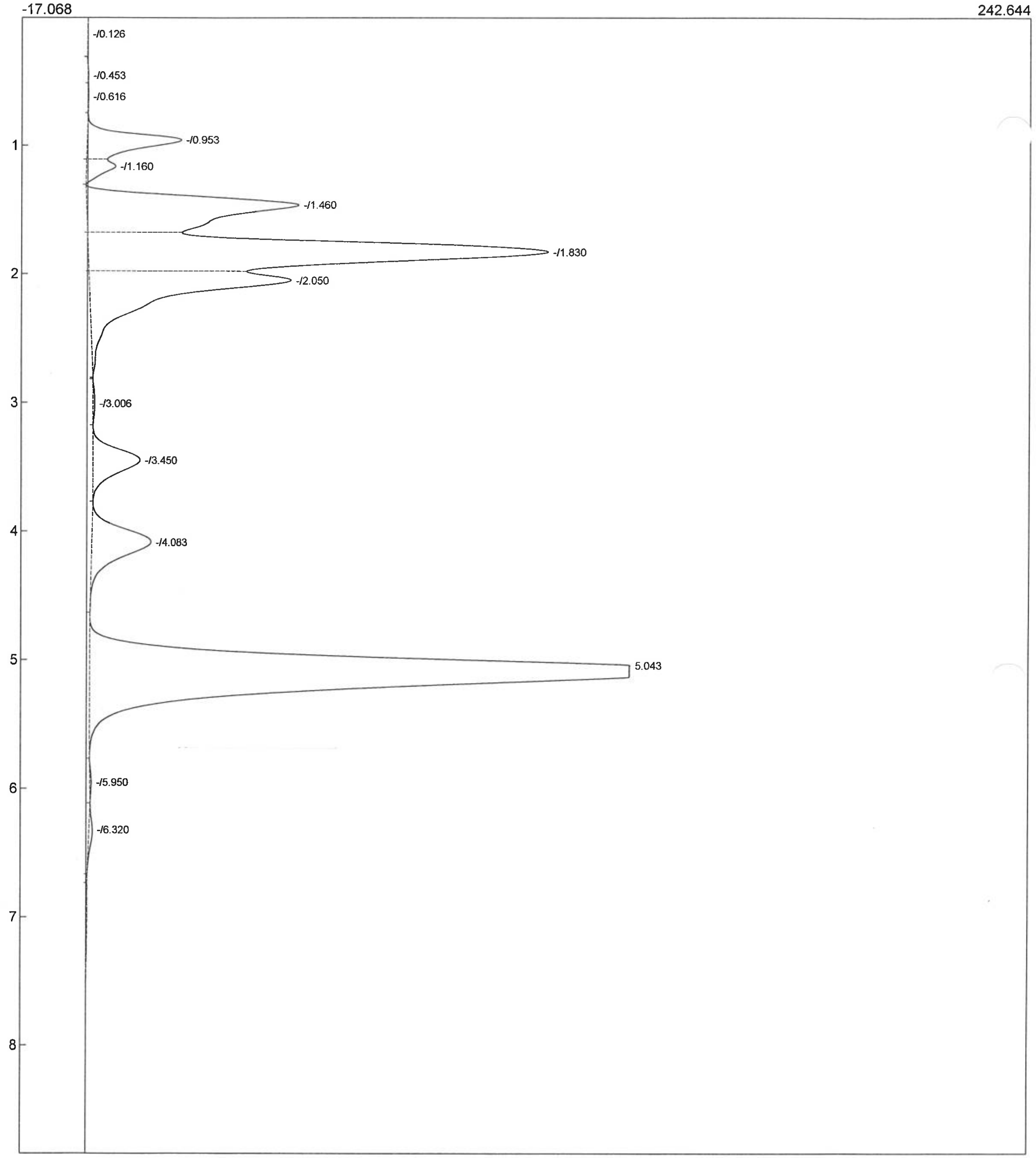

Component	Retention	Area	External	Units
	5.043	2355.9921	0.0000	
		2355.9921	0.0000	

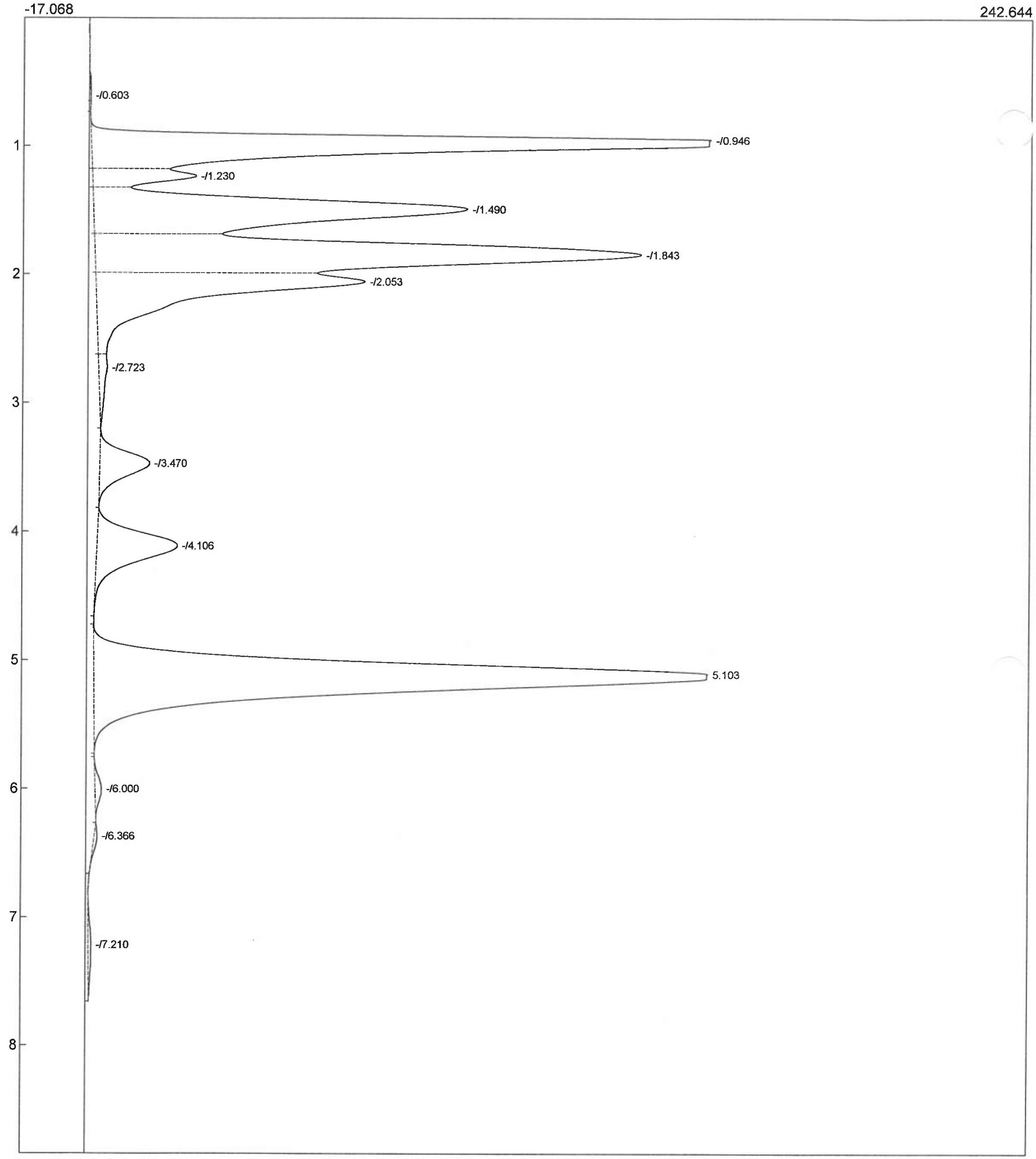

Component	Retention	Area	External	Units
	5.103	2482.7638	0.0000	
		2482.7638	0.0000	

9/18/18 191

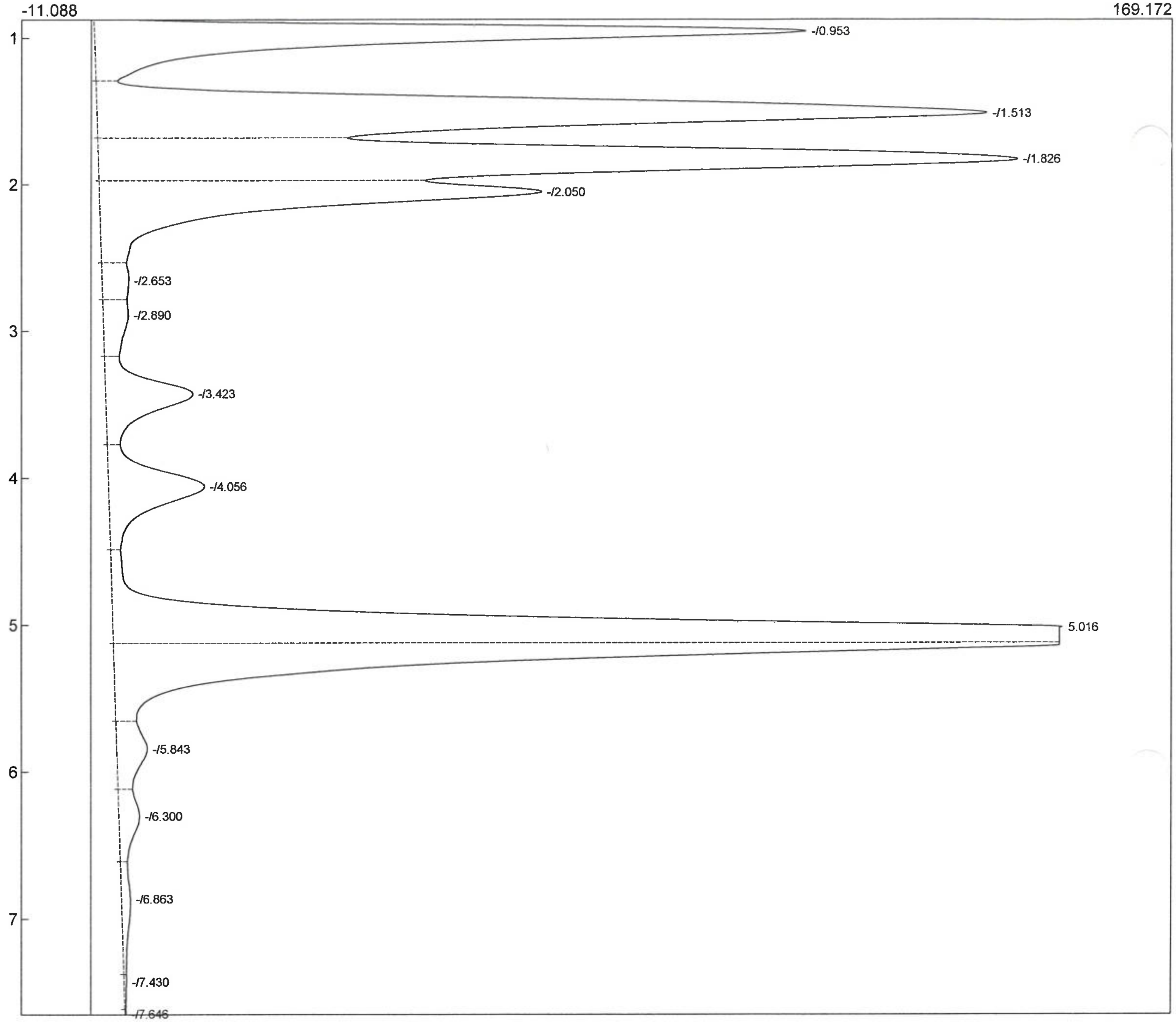

Component	Retention	Area	External	Units
	5.016	1670.2670	0.0000	
	0.000	0.0000	0.0000	
		1670.2670	0.0000	

Flat topped
re-run

162

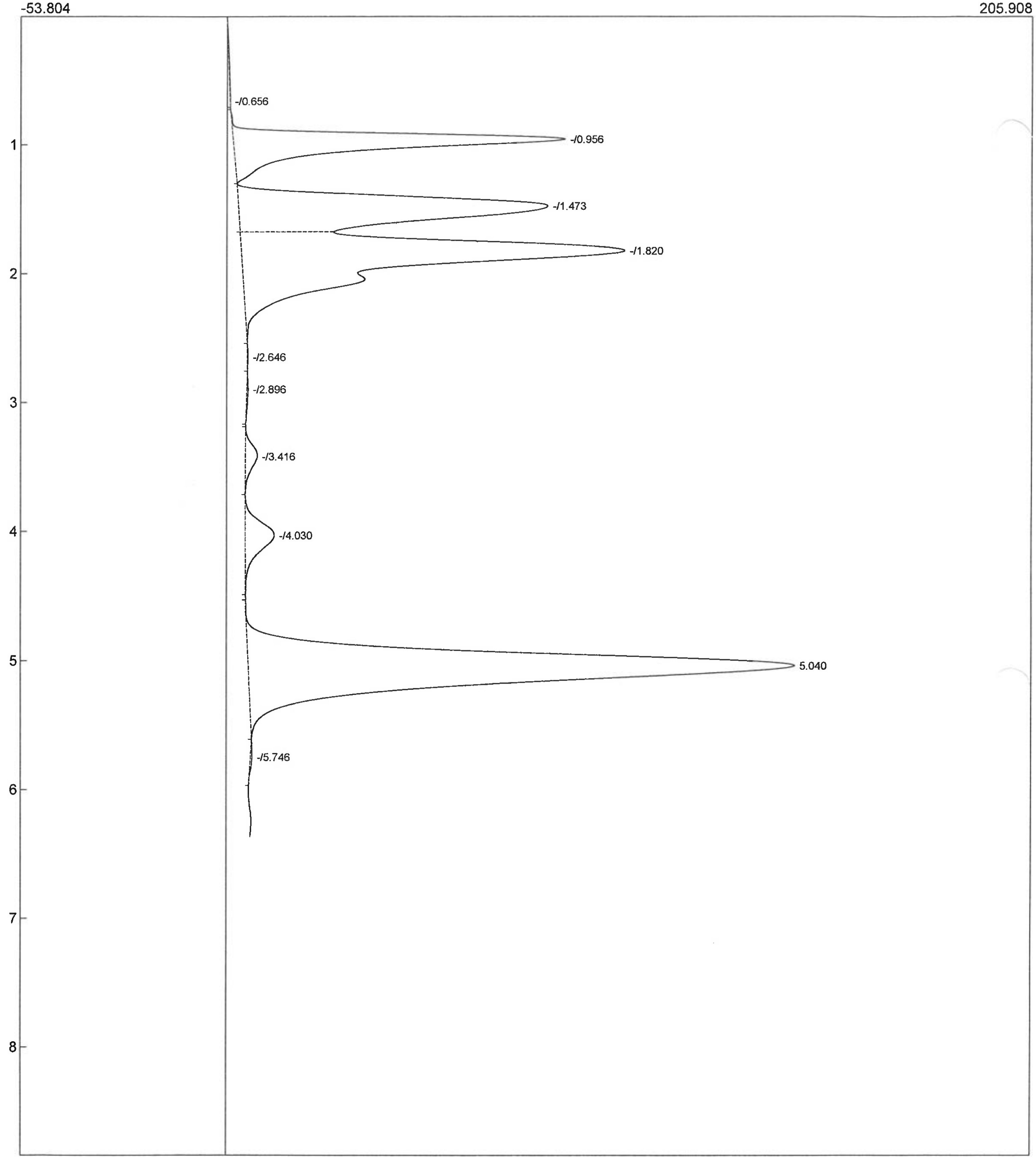

Component	Retention	Area	External	Units
	5.040	2201.2300	0.0000	
		2201.2300	0.0000	

F7 63
7/18/14

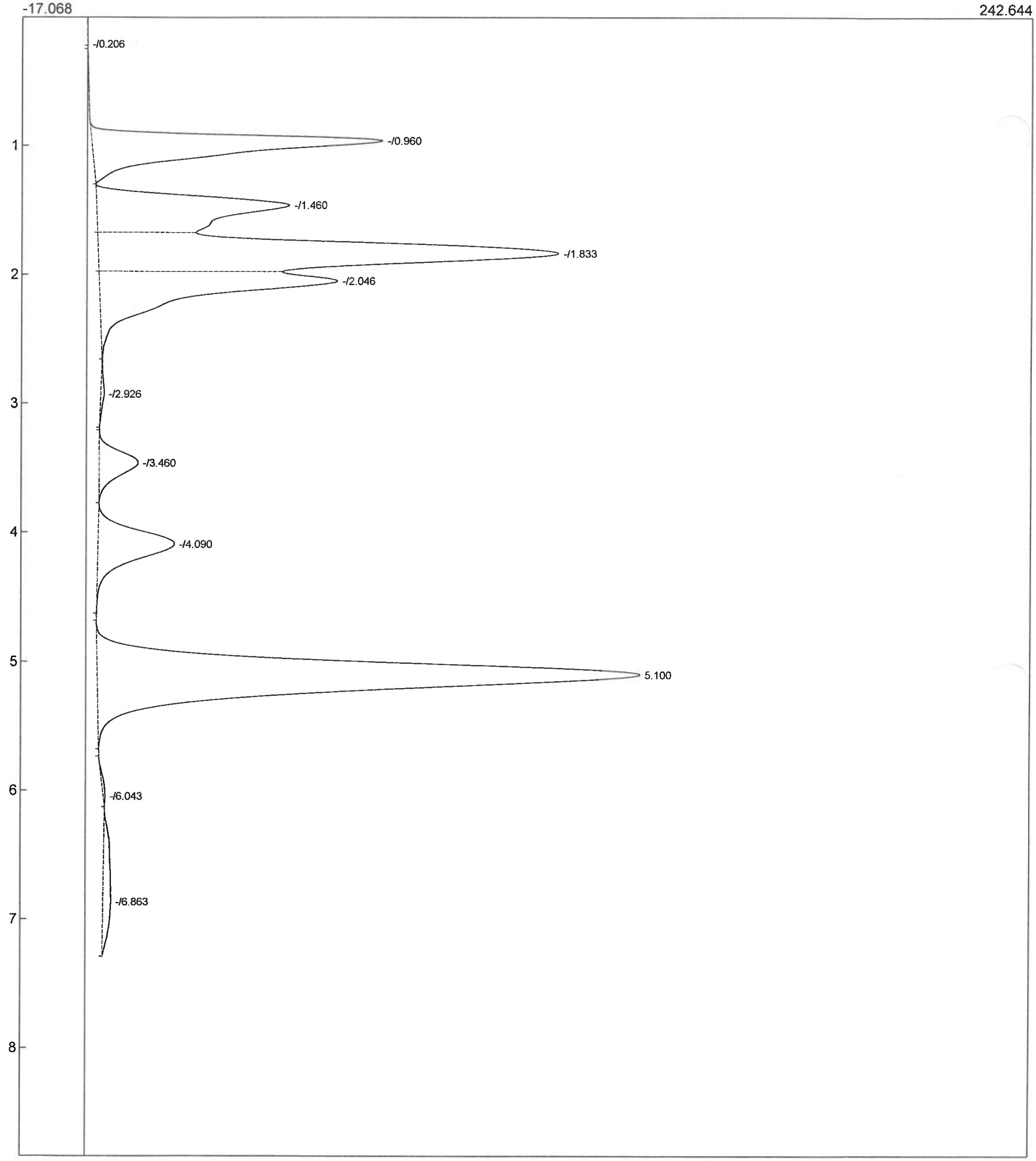

Component	Retention	Area	External	Units
	5.100	2106.2743	0.0000	
		2106.2743	0.0000	

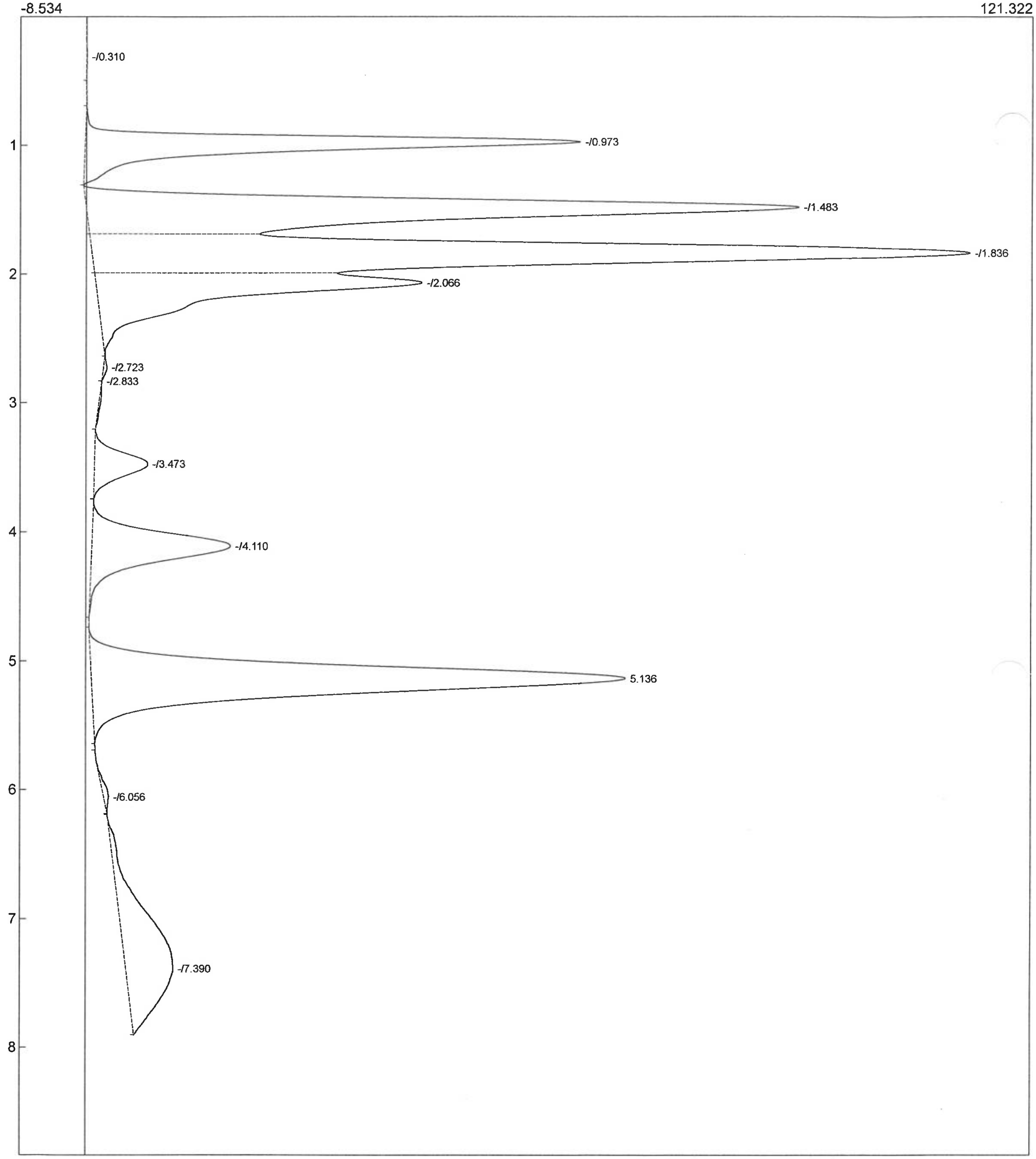

Component	Retention	Area	External	Units
	5.136	1009.6142	0.0000	
		1009.6142	0.0000	

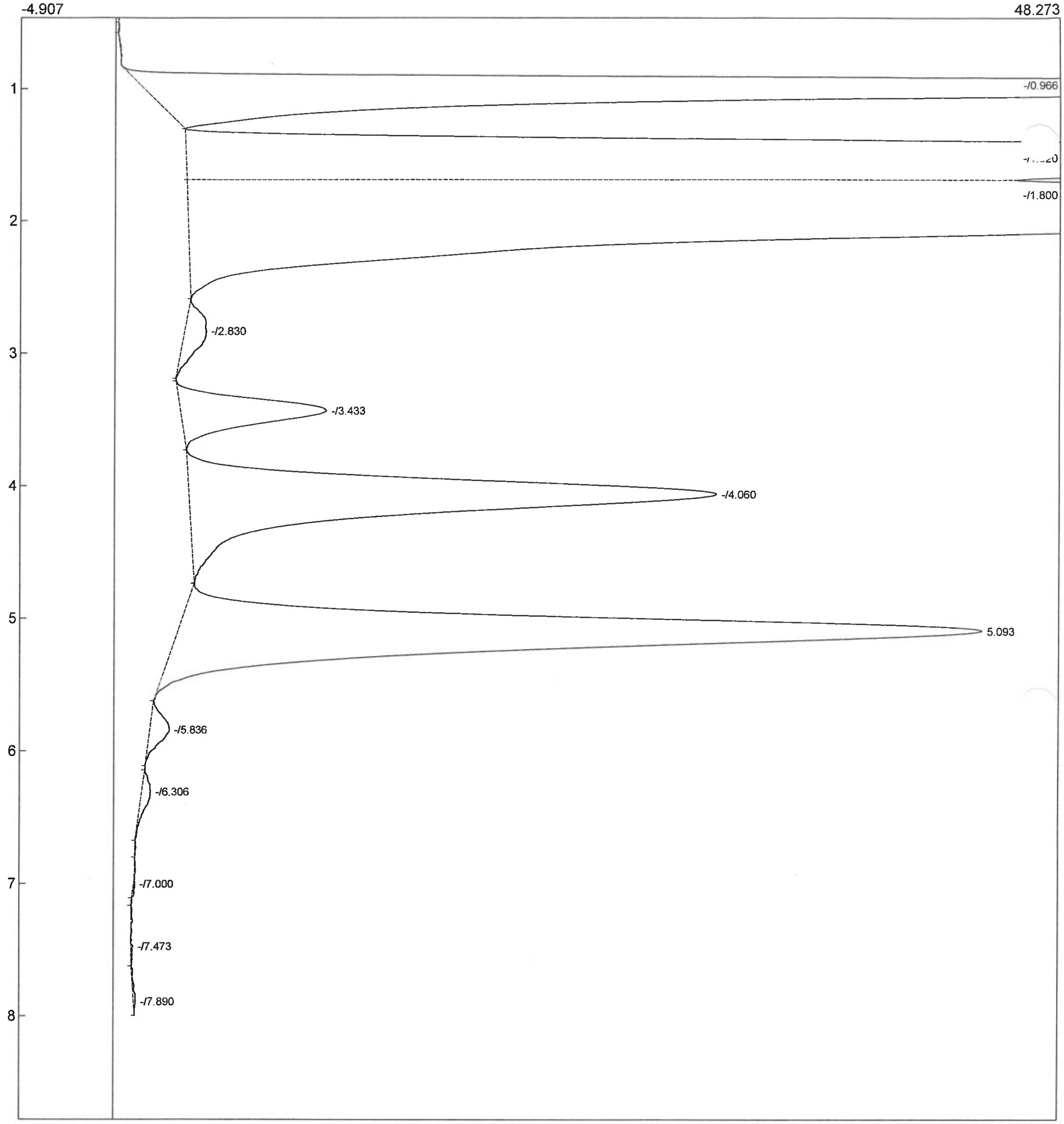

Component	Retention	Area	External	Units
	5.093	640.3717	0.0000	
		640.3717	0.0000	

7/18/18

166

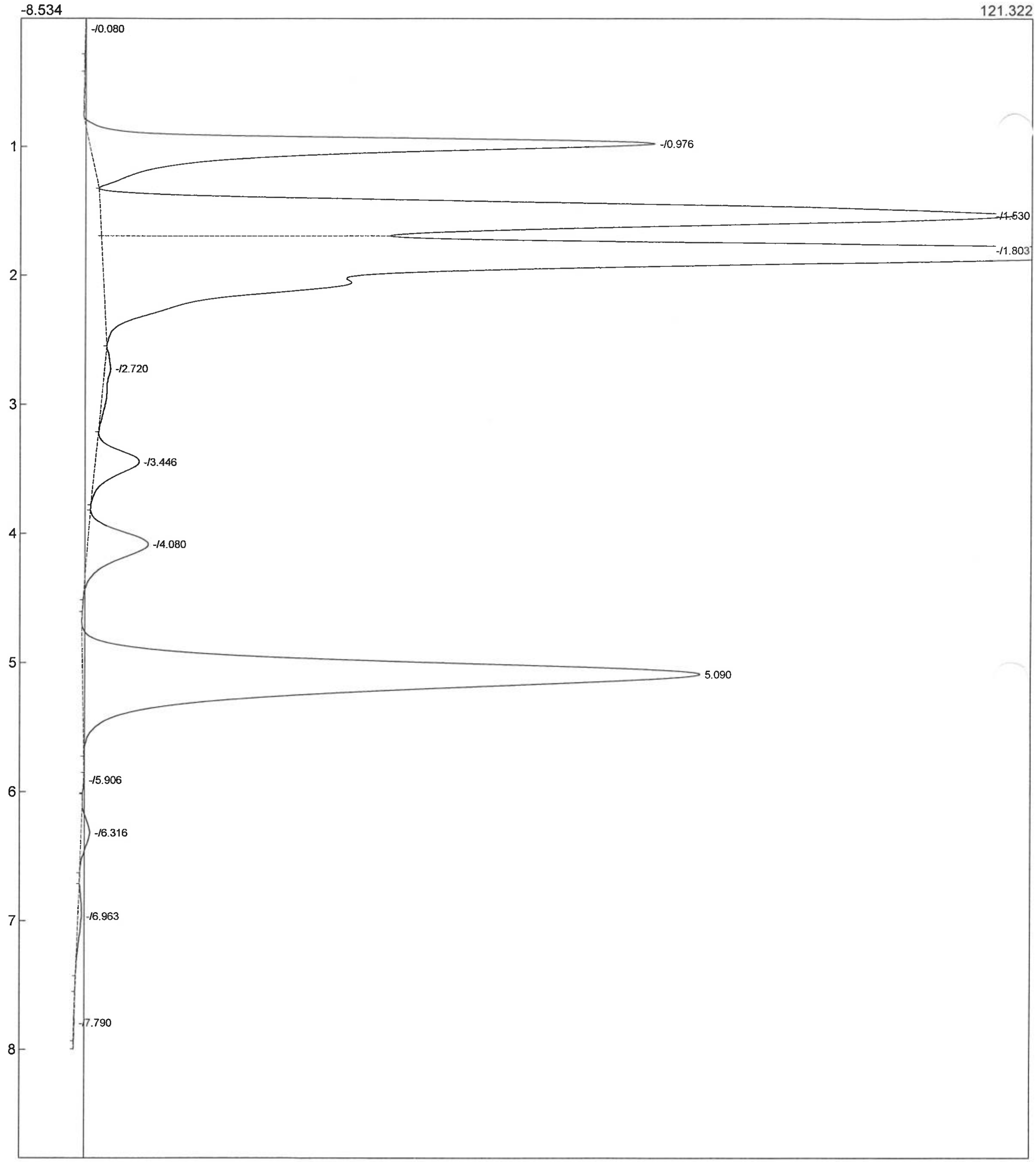

Component	Retention	Area	External	Units
	5.090	1227.9250	0.0000	
		1227.9250	0.0000	

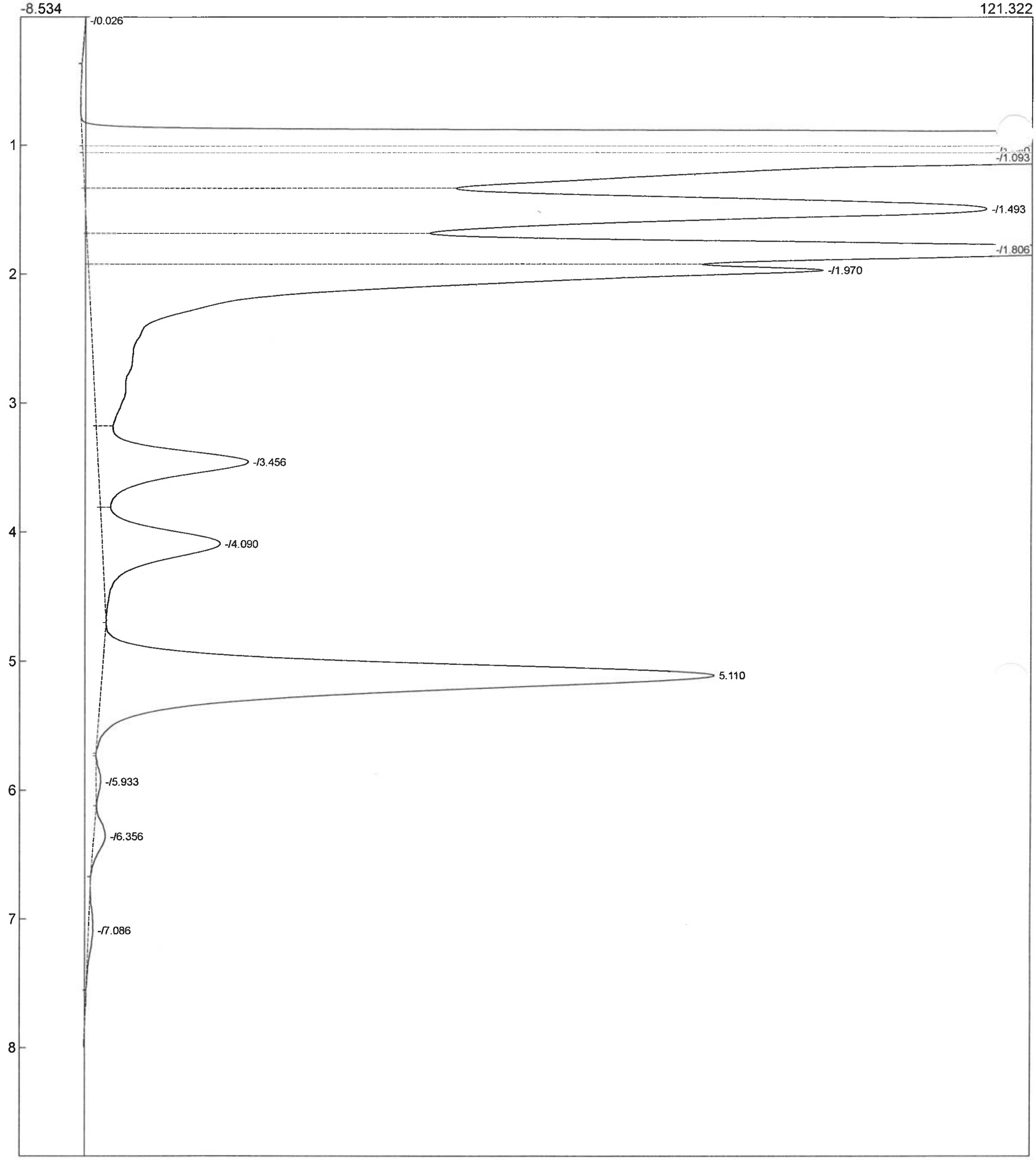

Component	Retention	Area	External	Units
	5.110	1213.8652	0.0000	
		1213.8652	0.0000	

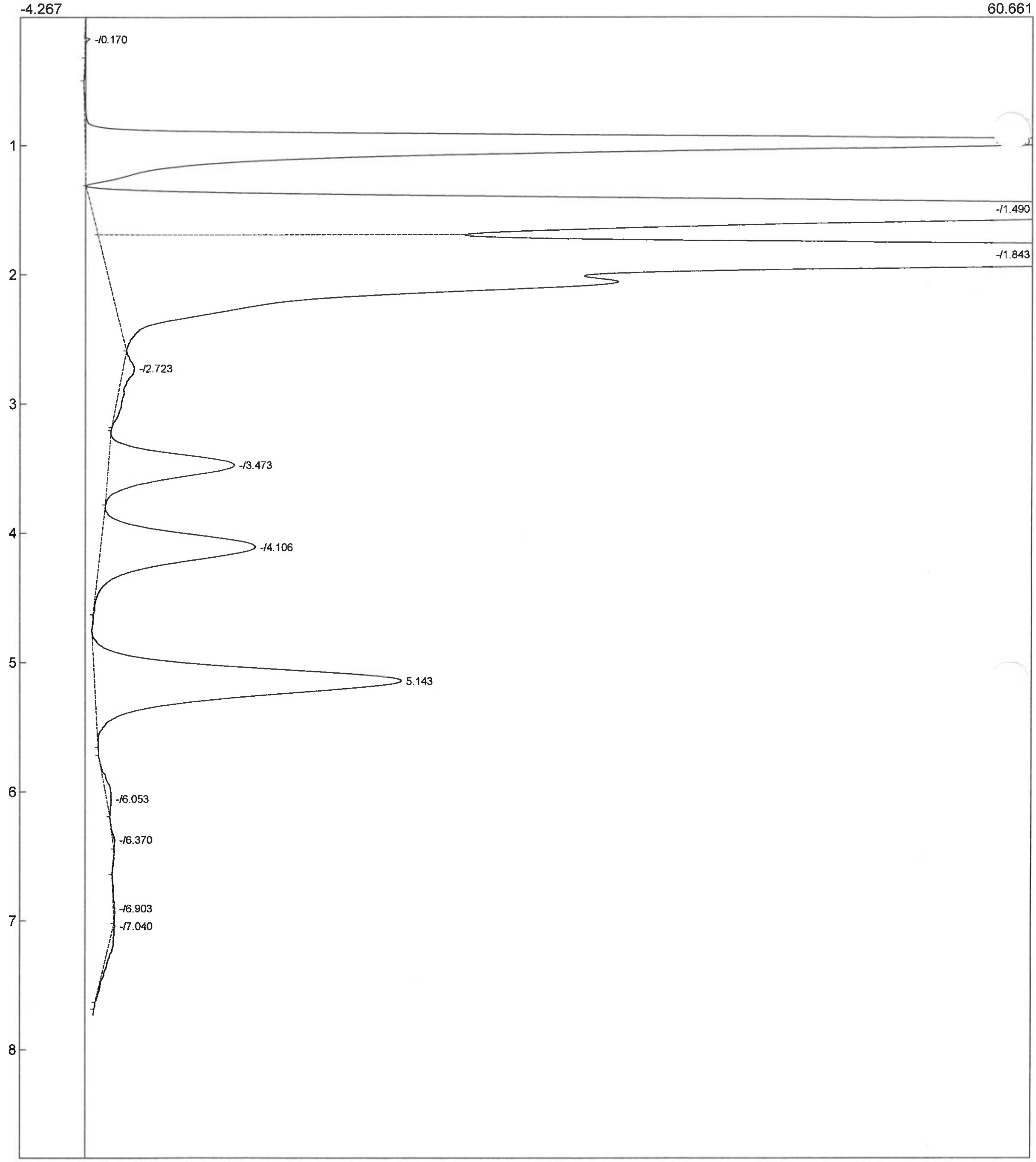

Component	Retention	Area	External	Units
	5.143	302.4406	0.0000	
		302.4406	0.0000	

7/18/18

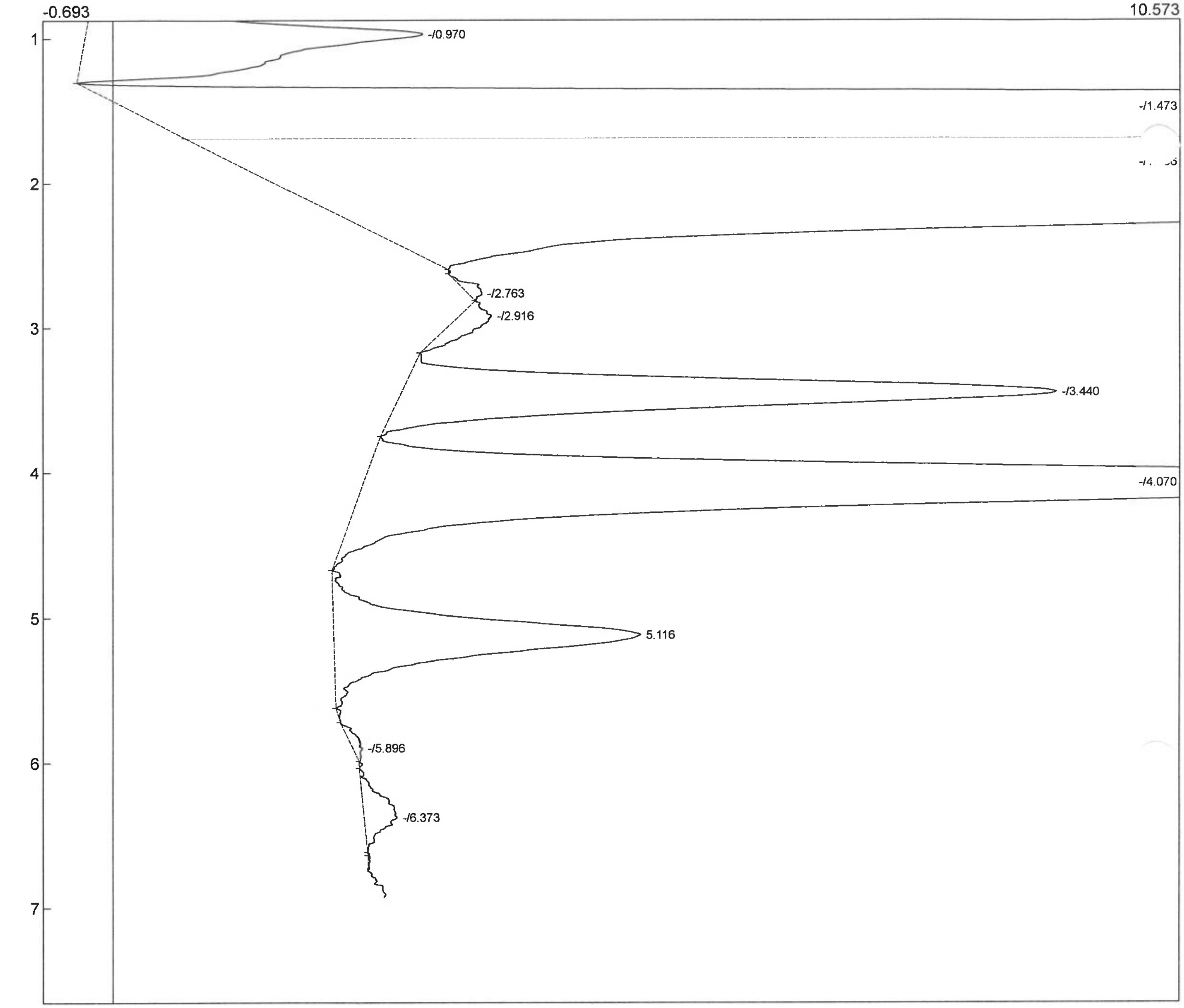

Component	Retention	Area	External	Units
-	0.970	79.4154	0.0000	
-	1.473	1052.3591	0.0000	
-	1.856	1772.8083	0.0000	
-	2.763	1.0305	0.0000	
-	2.916	4.5285	0.0000	
-	3.440	81.6050	0.0000	
-	4.070	222.2967	0.0000	
	0.000	0.0000	0.0000	
	5.116	51.6115	0.0000	
-	5.896	1.0548	0.0000	
-	6.373	5.2859	0.0000	
		3271.9957	0.0000	

Fish 170
7/18/2018
1337 psi
R 990 0.0005

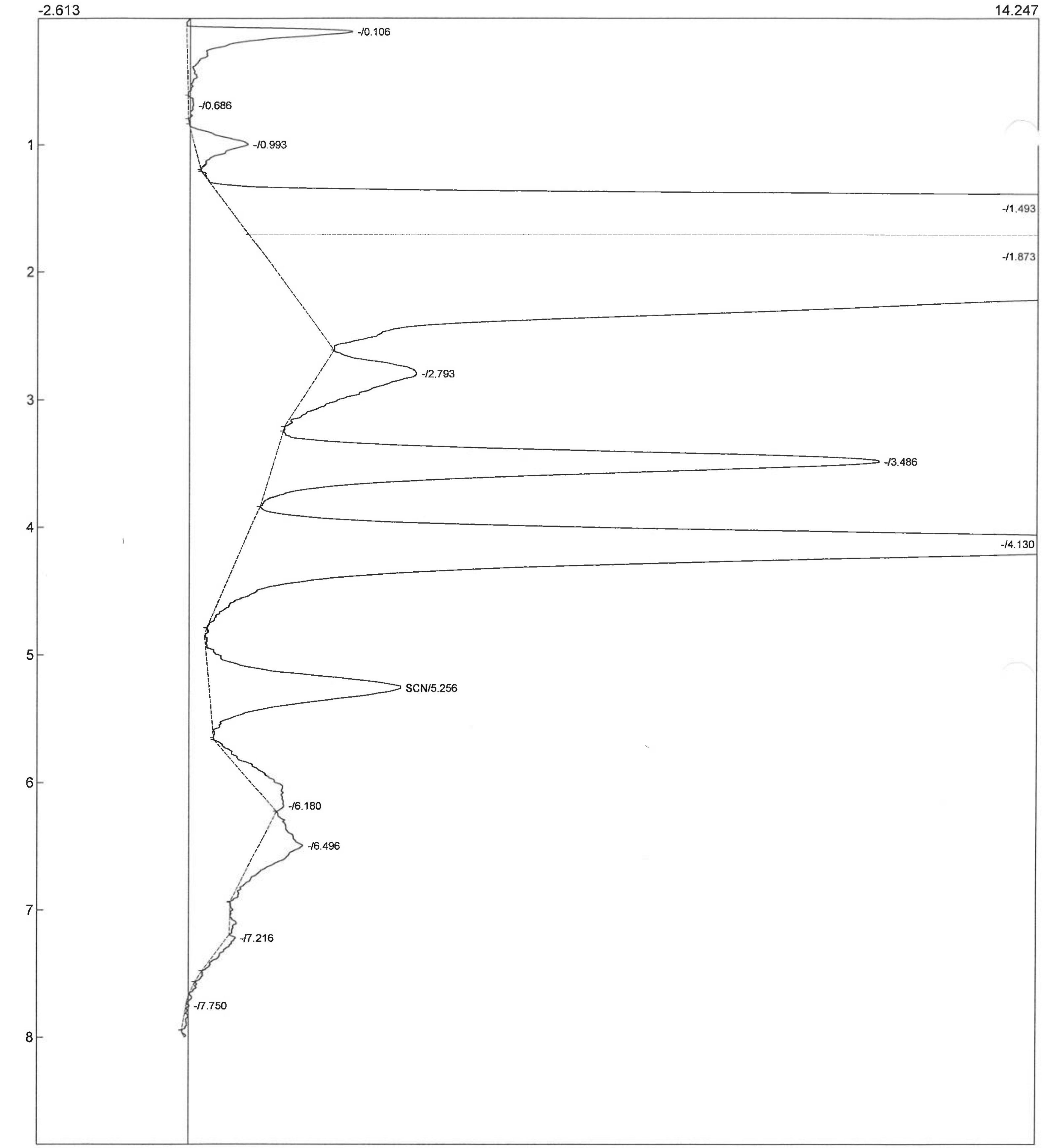

Component	Retention	Area	External	Units
SCN	5.256	47.7914	0.0000	
		47.7914	0.0000	

1:0
Run 2
1270/S:
7/23/18

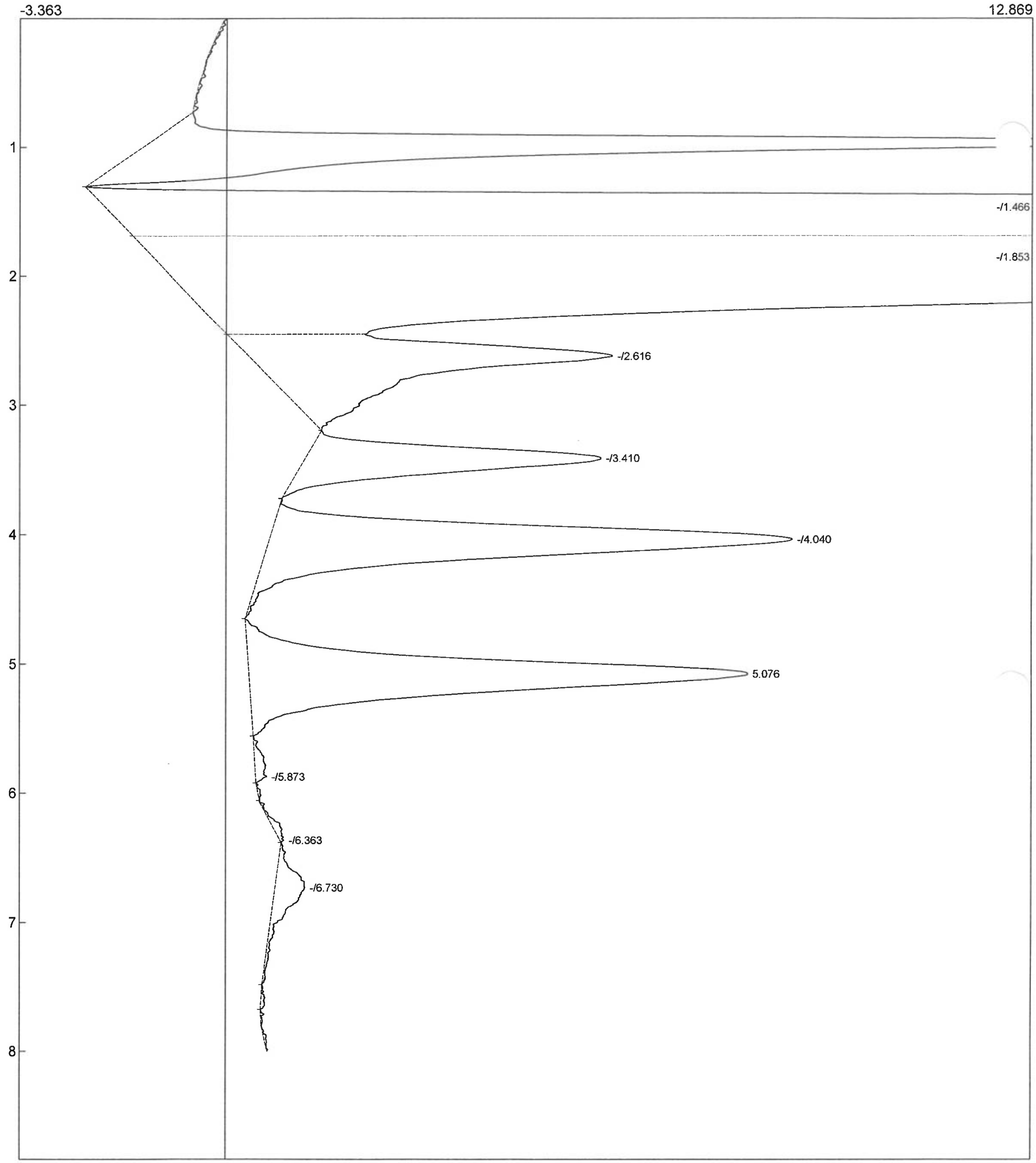

Component	Retention	Area	External	Units
	5.076	134.1116	0.0000	
		134.1116	0.0000	

F171
7/18/18

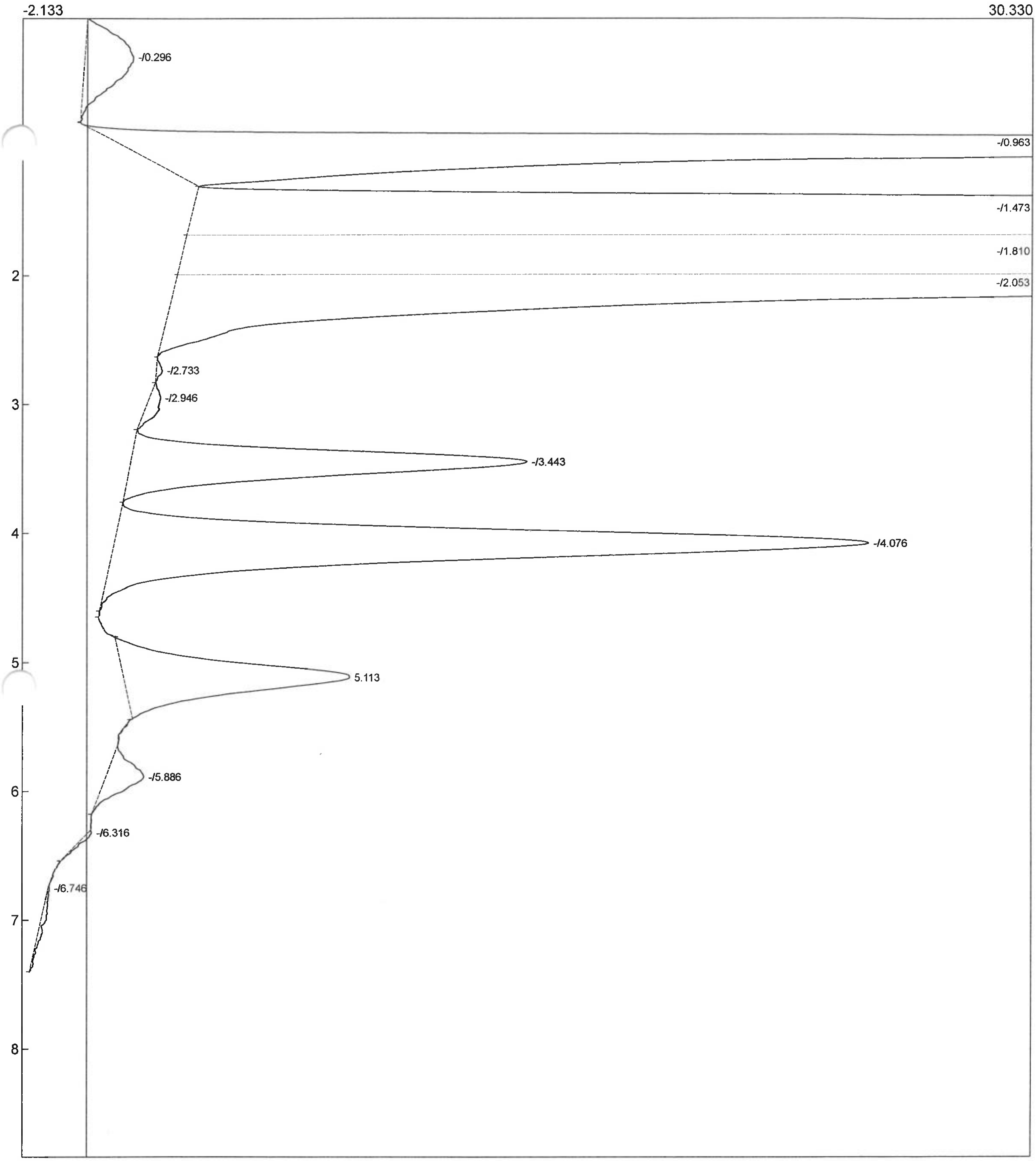

Component	Retention	Area	External	Units
	5.113	115.8720	0.0000	
		115.8720	0.0000	

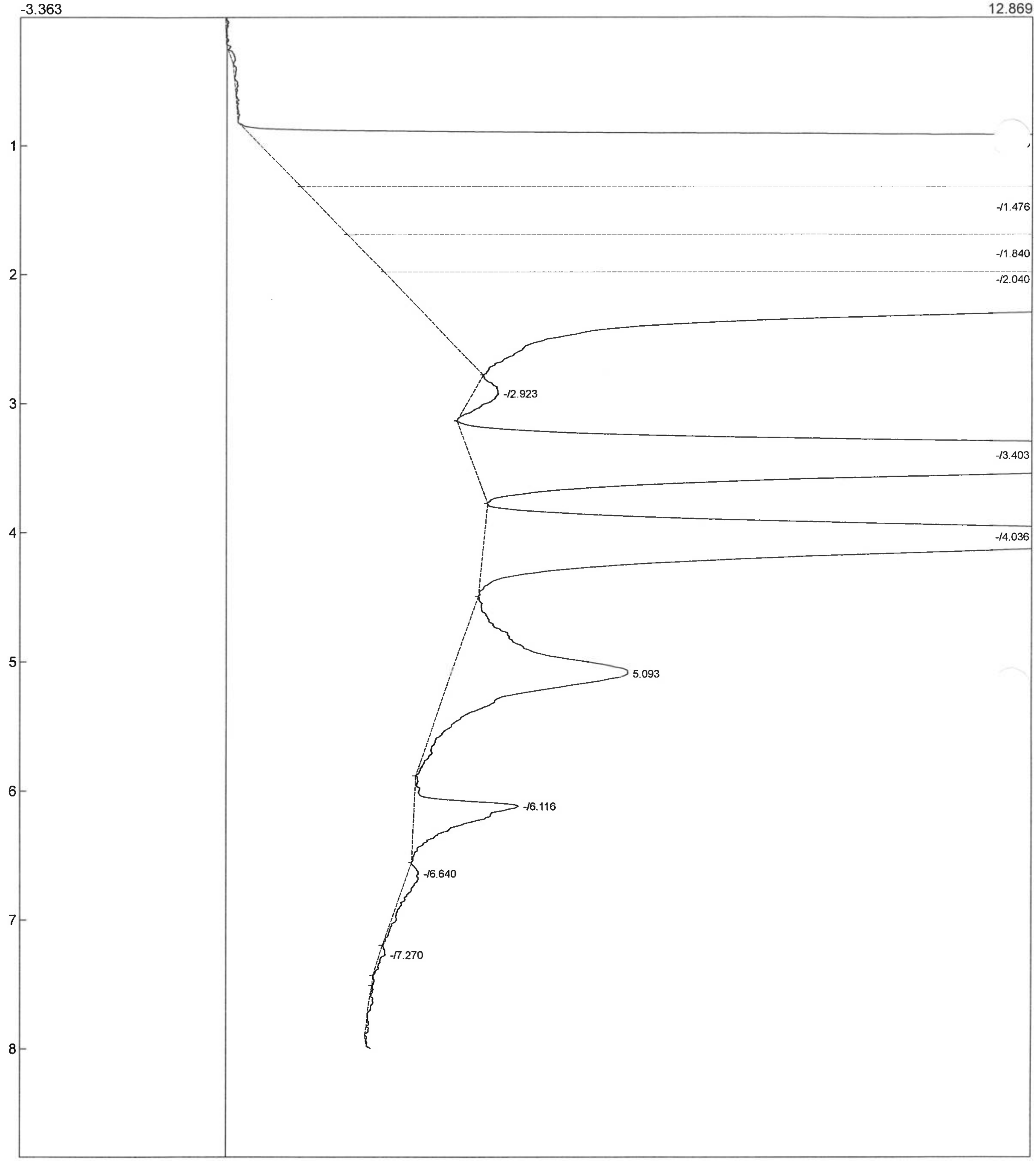

Component	Retention	Area	External	Units
	5.093	65.1629	0.0000	
		65.1629	0.0000	

173

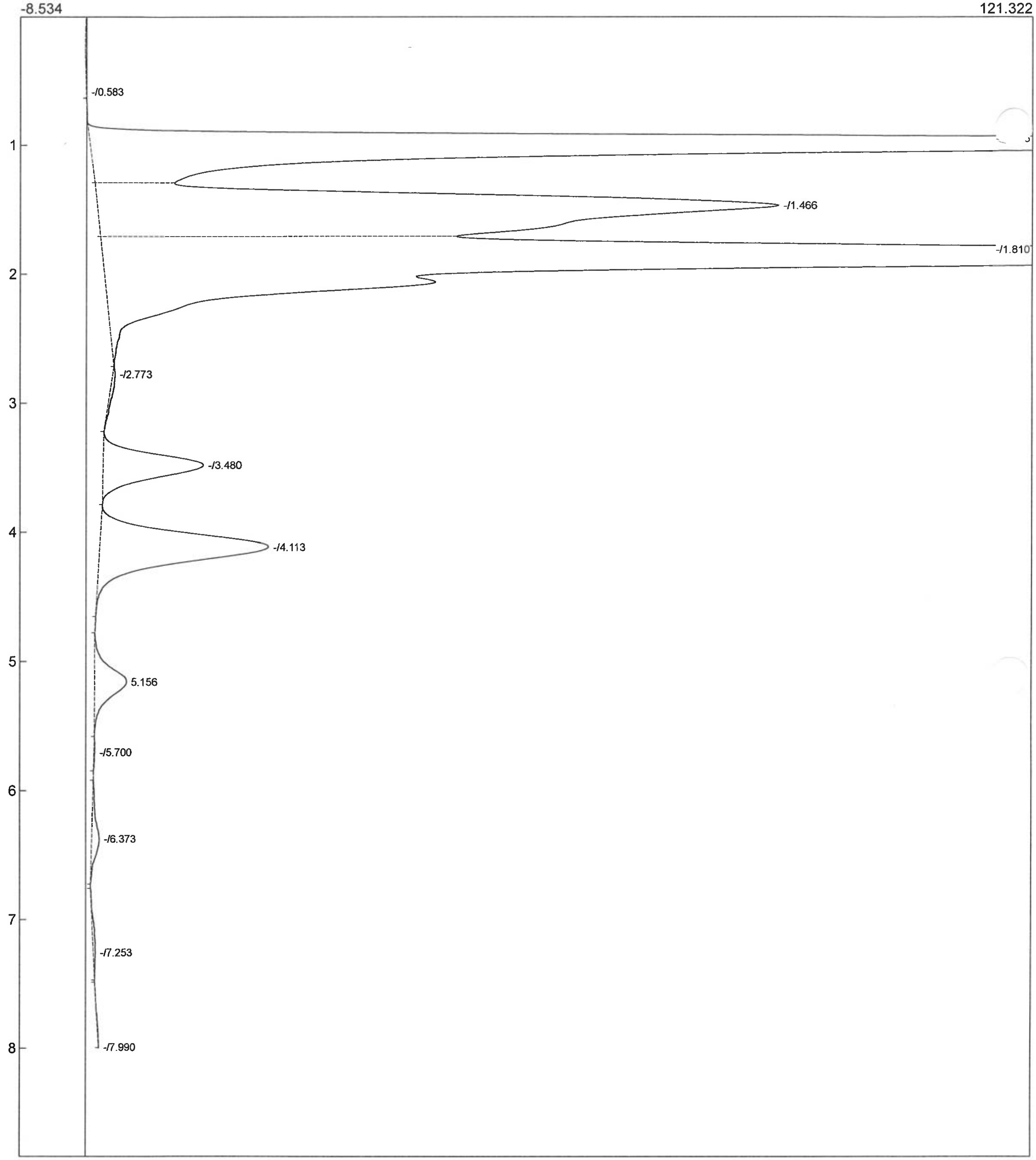

Component	Retention	Area	External	Units
	5.156	66.8688	0.0000	
		66.8688	0.0000	

FCI174
6/18/18

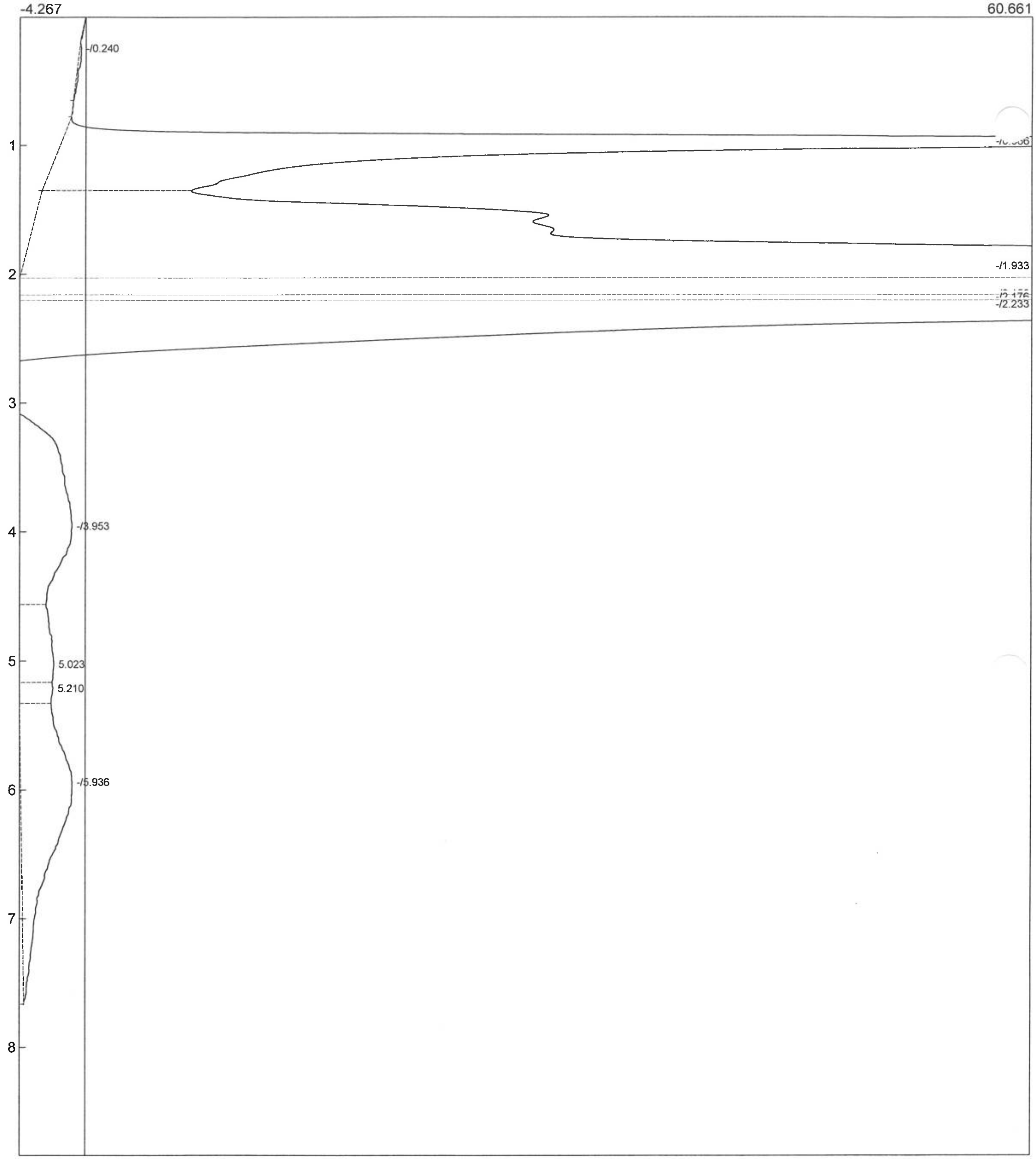

Component	Retention	Area	External	Units
	5.023	139.2164	0.0000	
	5.210	35.2697	0.0000	
		174.4861	0.0000	

1+G
4/18/18

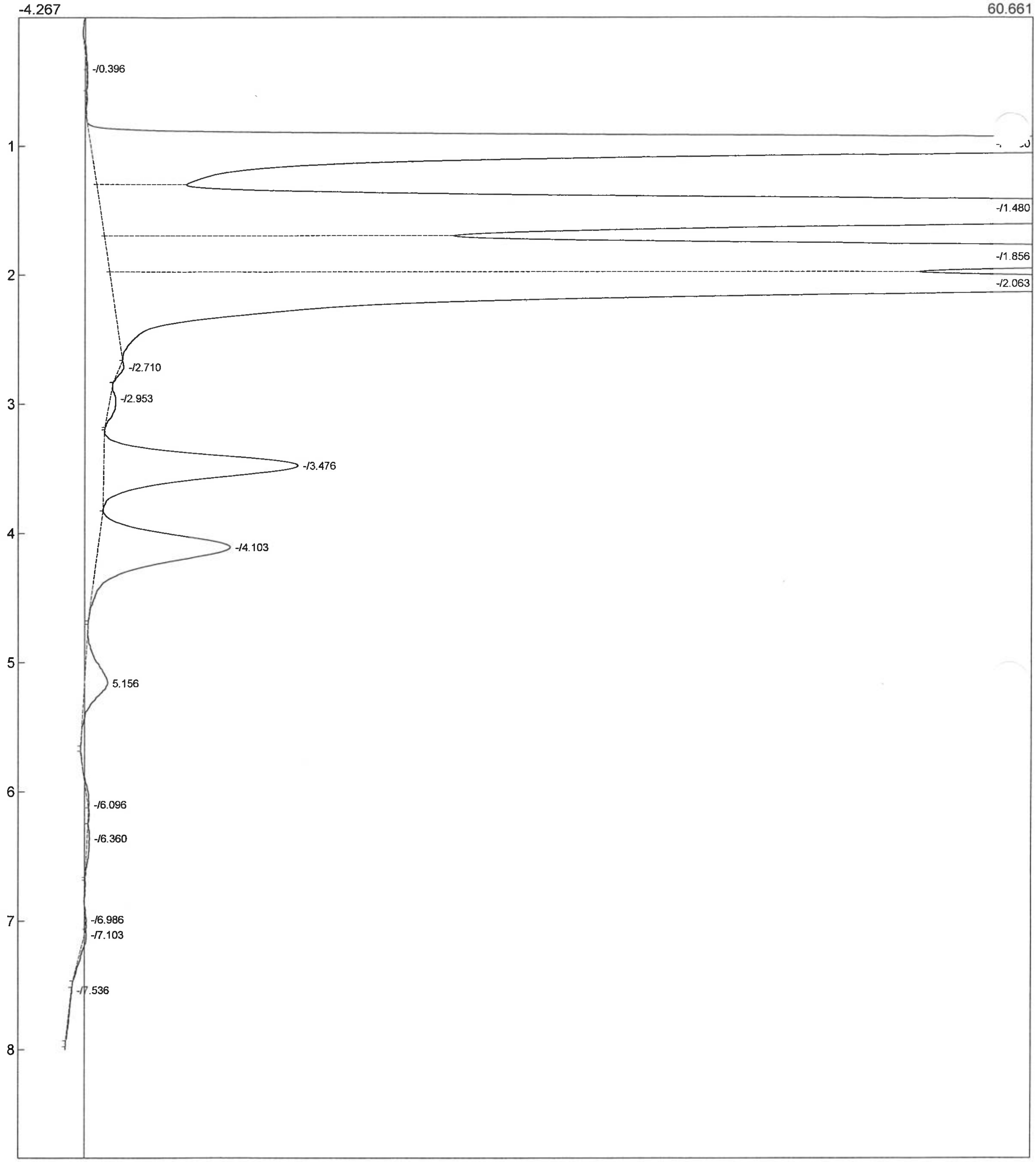

Component	Retention	Area	External	Units
	5.156	28.2044	0.0000	
		28.2044	0.0000	

1.5 175
7/18/18
1230 15'

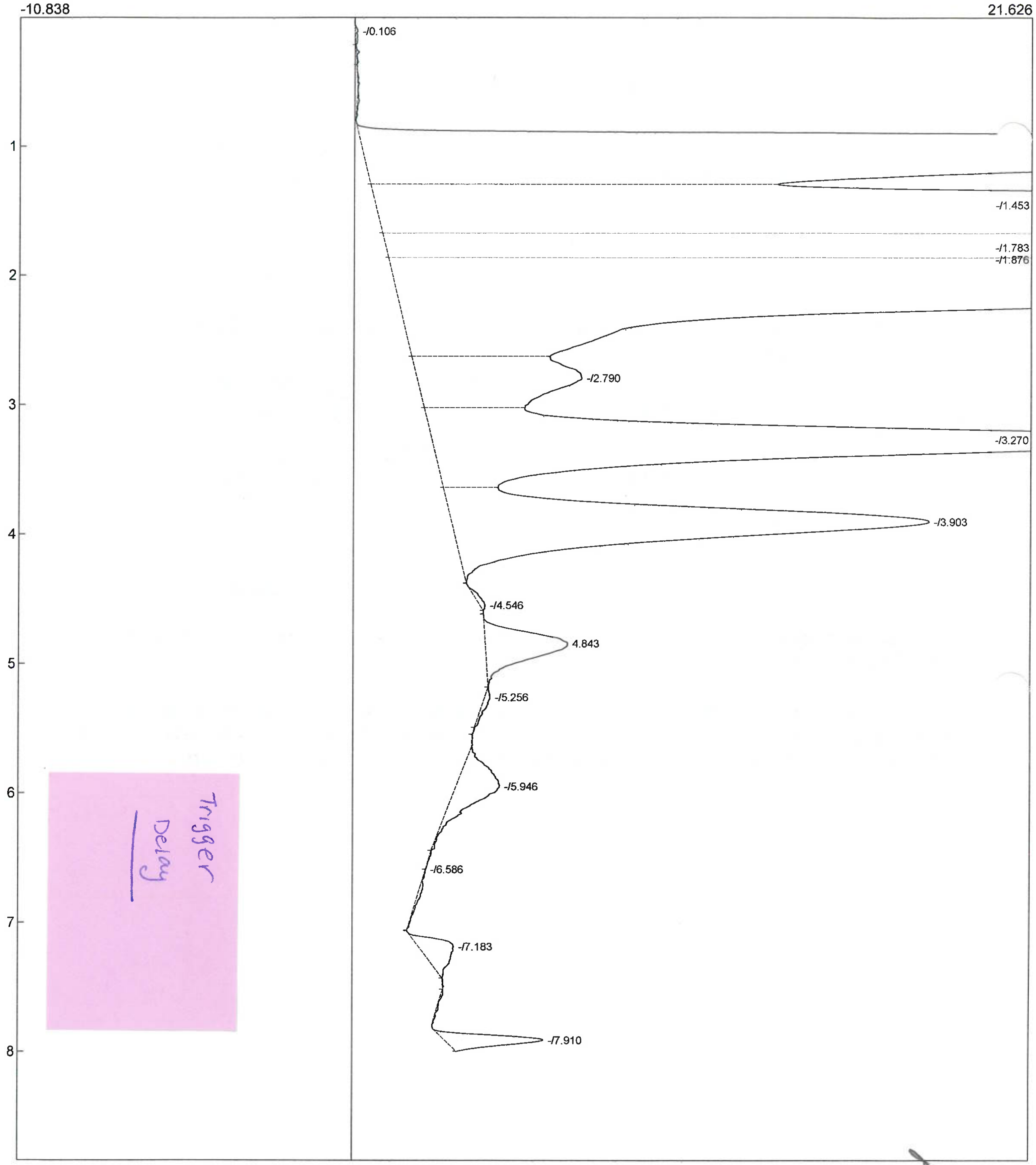

Component	Retention	Area	External	Units
	4.843	34.9802	0.0000	
		34.9802	0.0000	

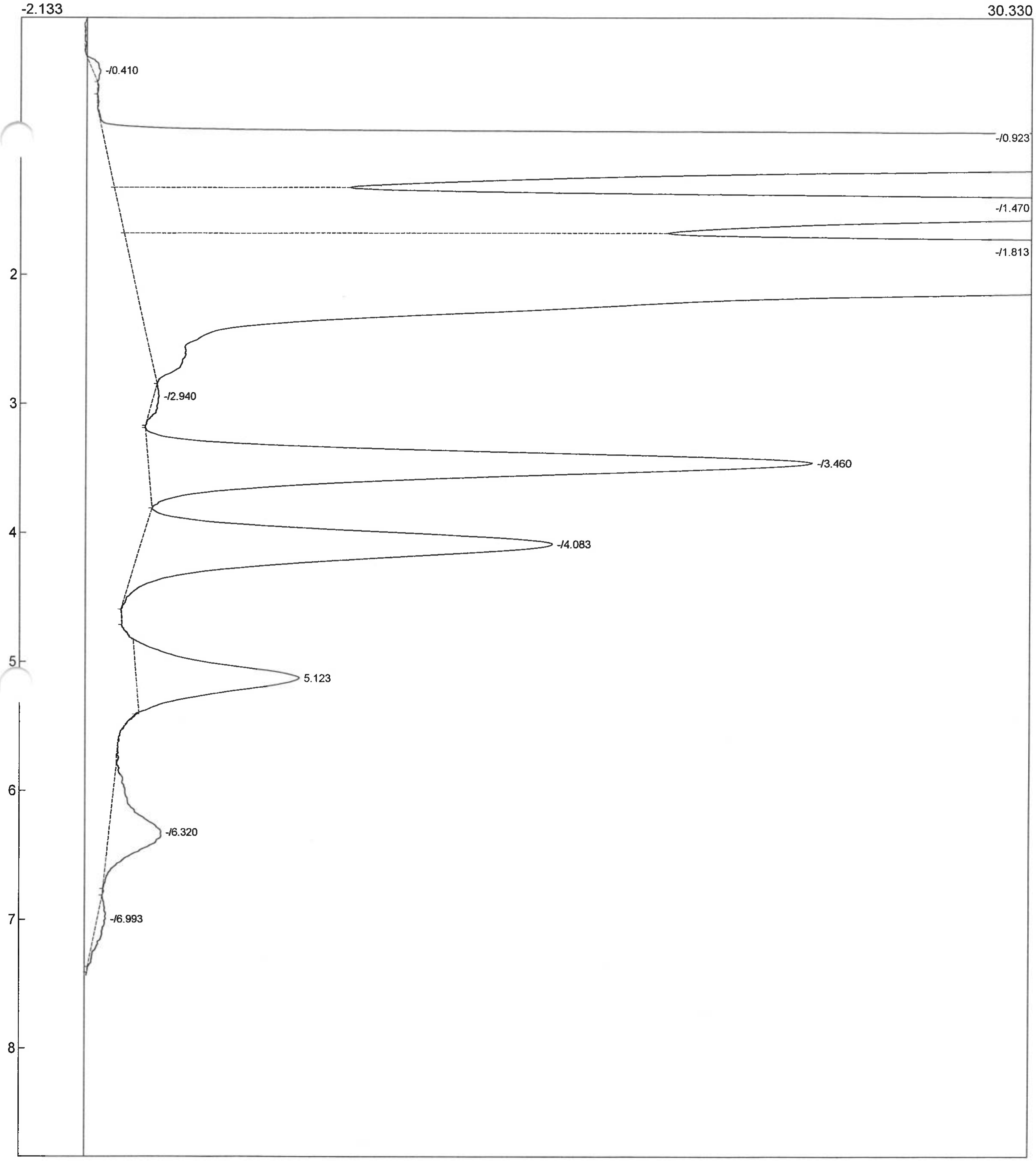

Component	Retention	Area	External	Units
	5.123	78.8017	0.0000	
		78.8017	0.0000	

8/18/15 [illegible]

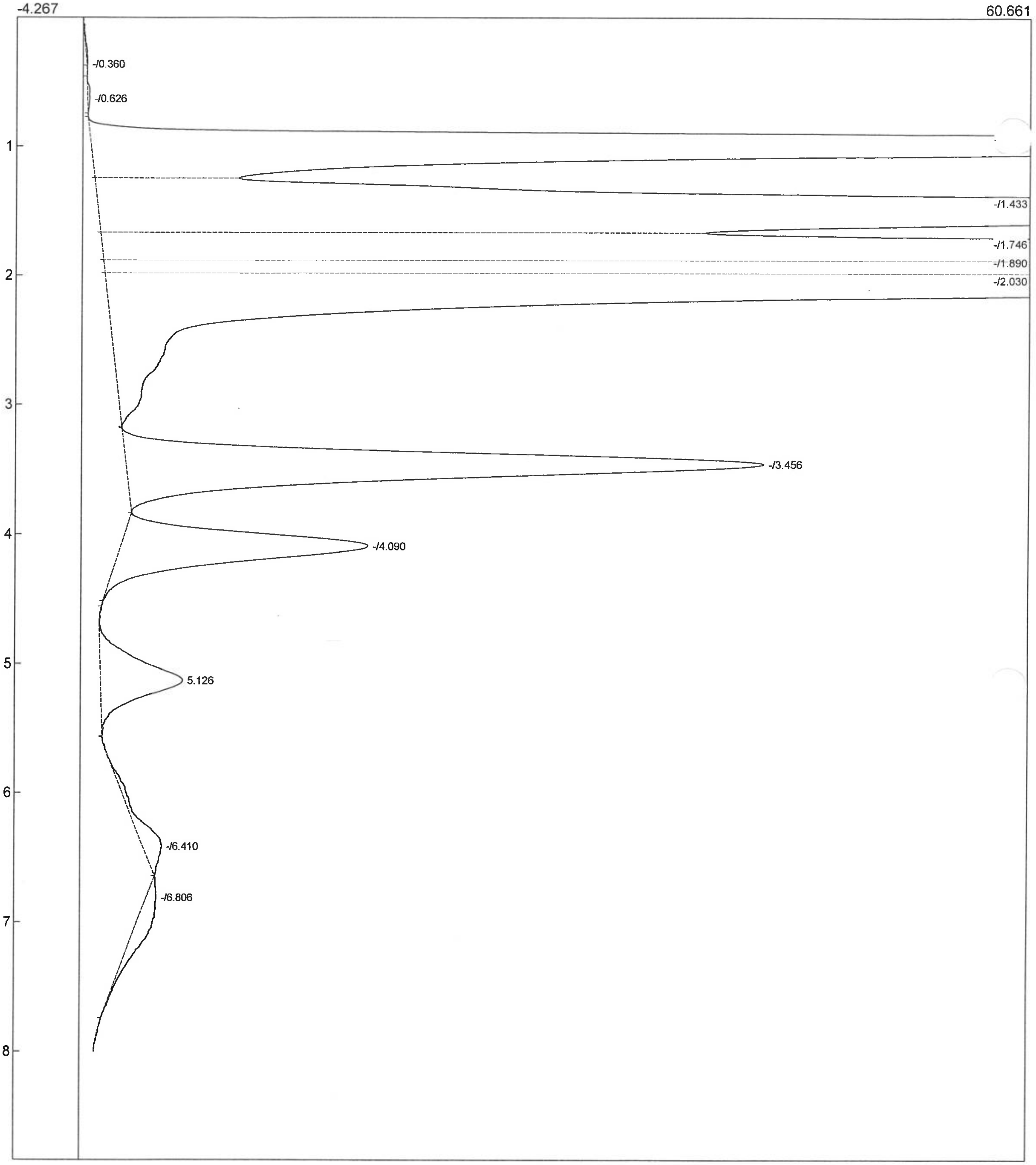

Component	Retention	Area	External	Units
	5.126	100.0215	0.0000	
		100.0215	0.0000	

F4174

7/18/16

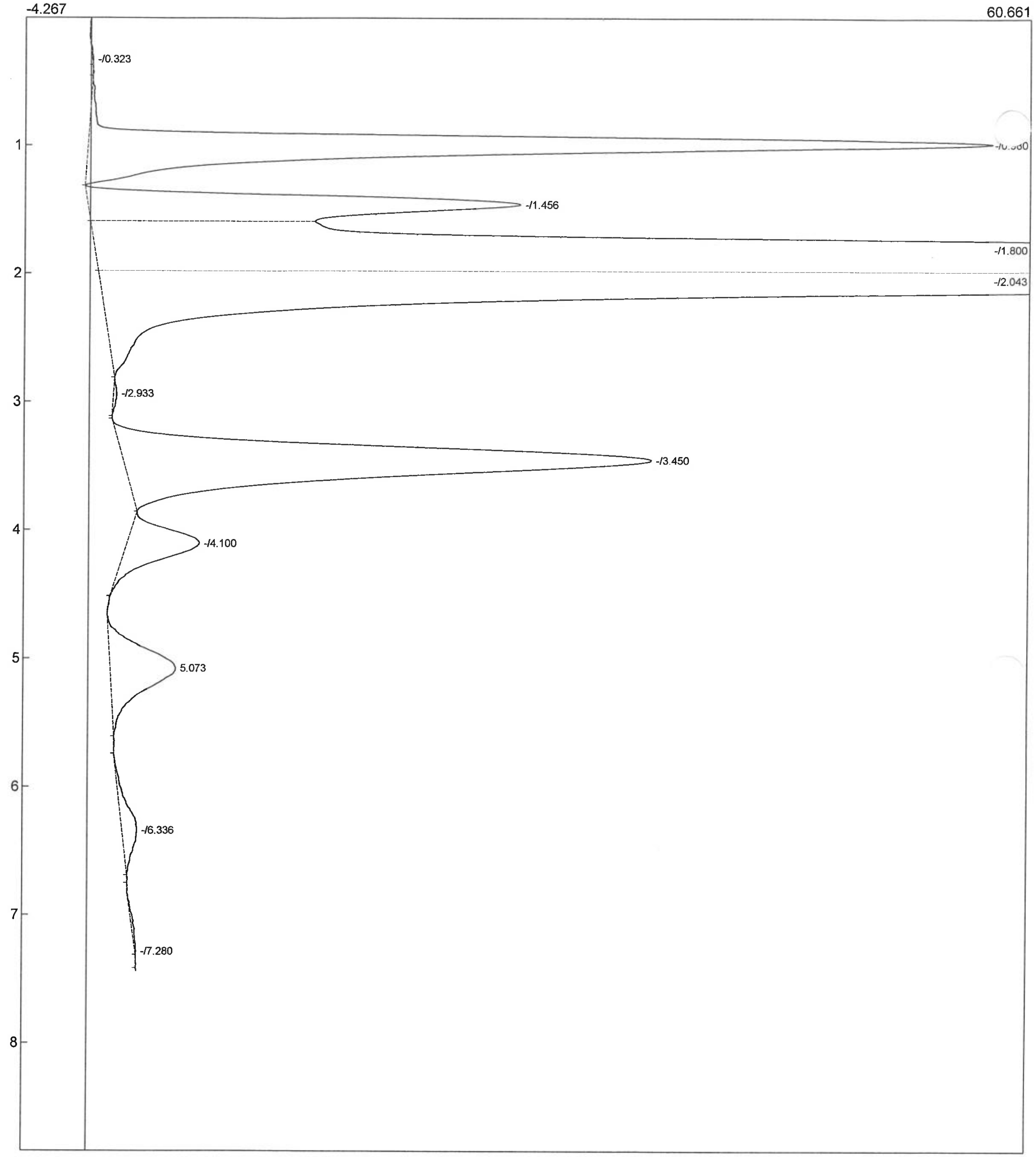

Component	Retention	Area	External	Units
	5.073	96.8868	0.0000	
		96.8868	0.0000	

7/18/18 160

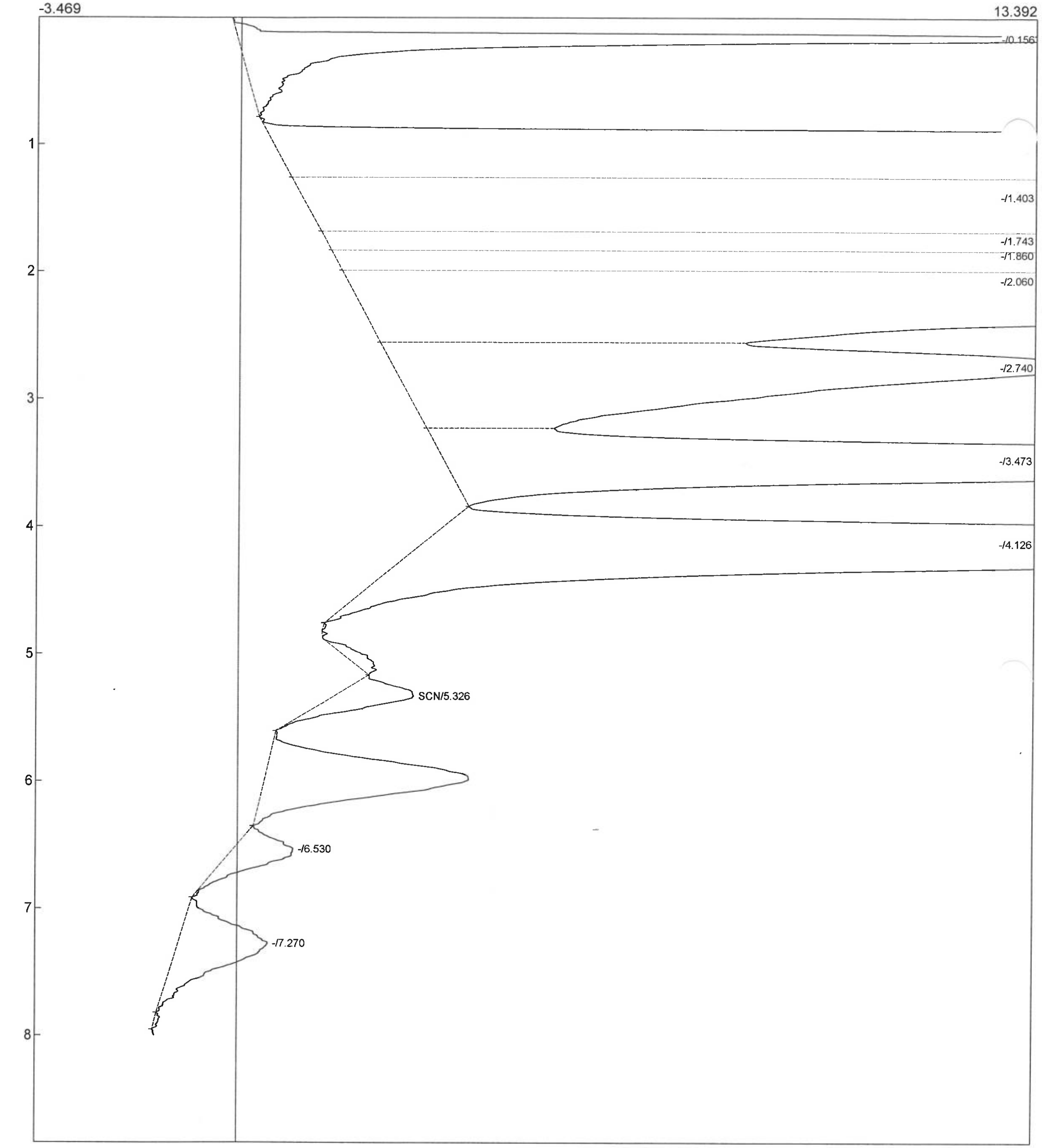

Component	Retention	Area	External	Units
SCN	5.326	82.9320	0.0000	
		82.9320	0.0000	

fish 181
72 hours
1290psi
7/23/18

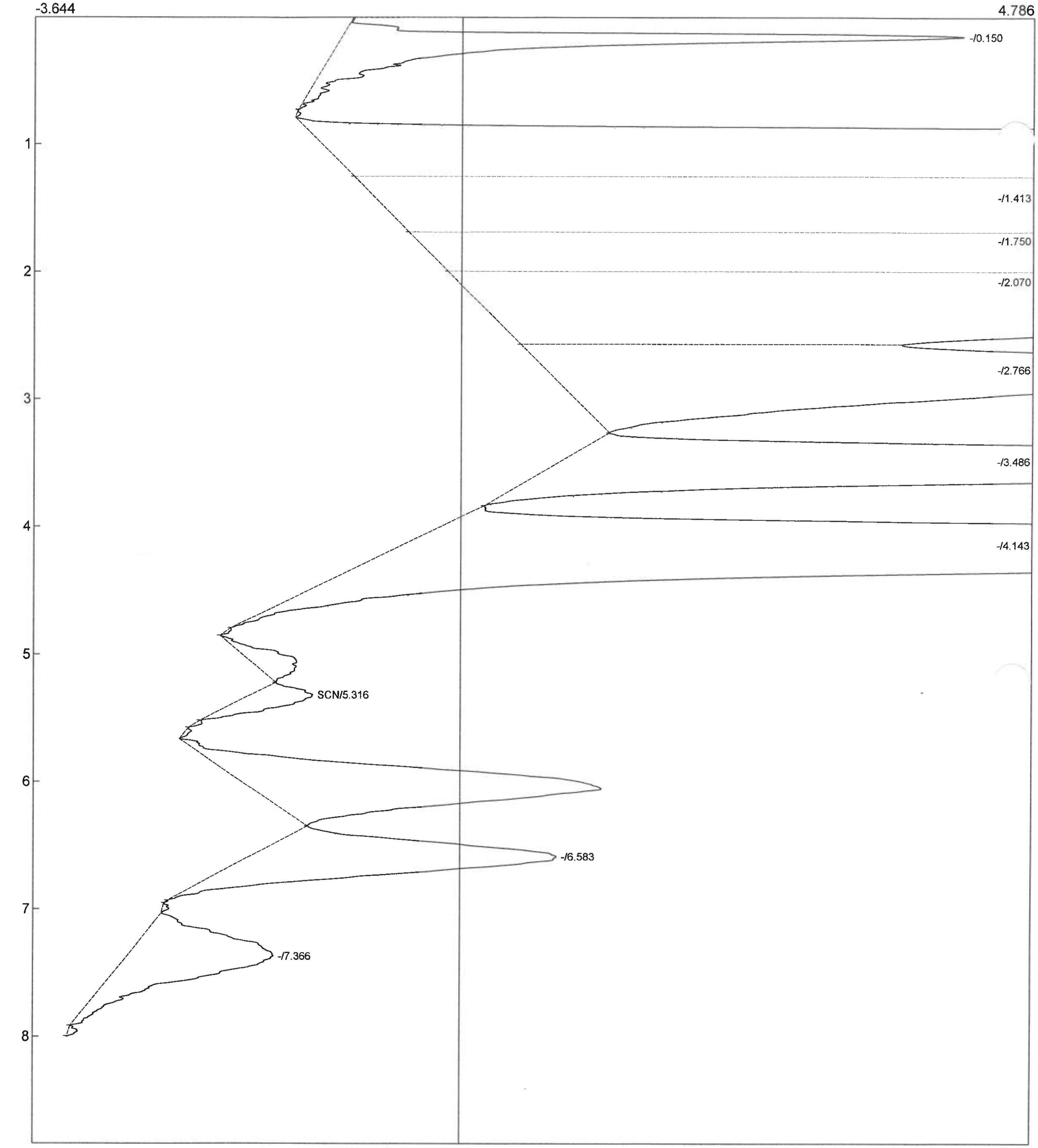

Component	Retention	Area	External	Units
SCN	5.316	60.9727	0.0000	
		60.9727	0.0000	

fish 182
72 hours
[illegible]
7/23/18

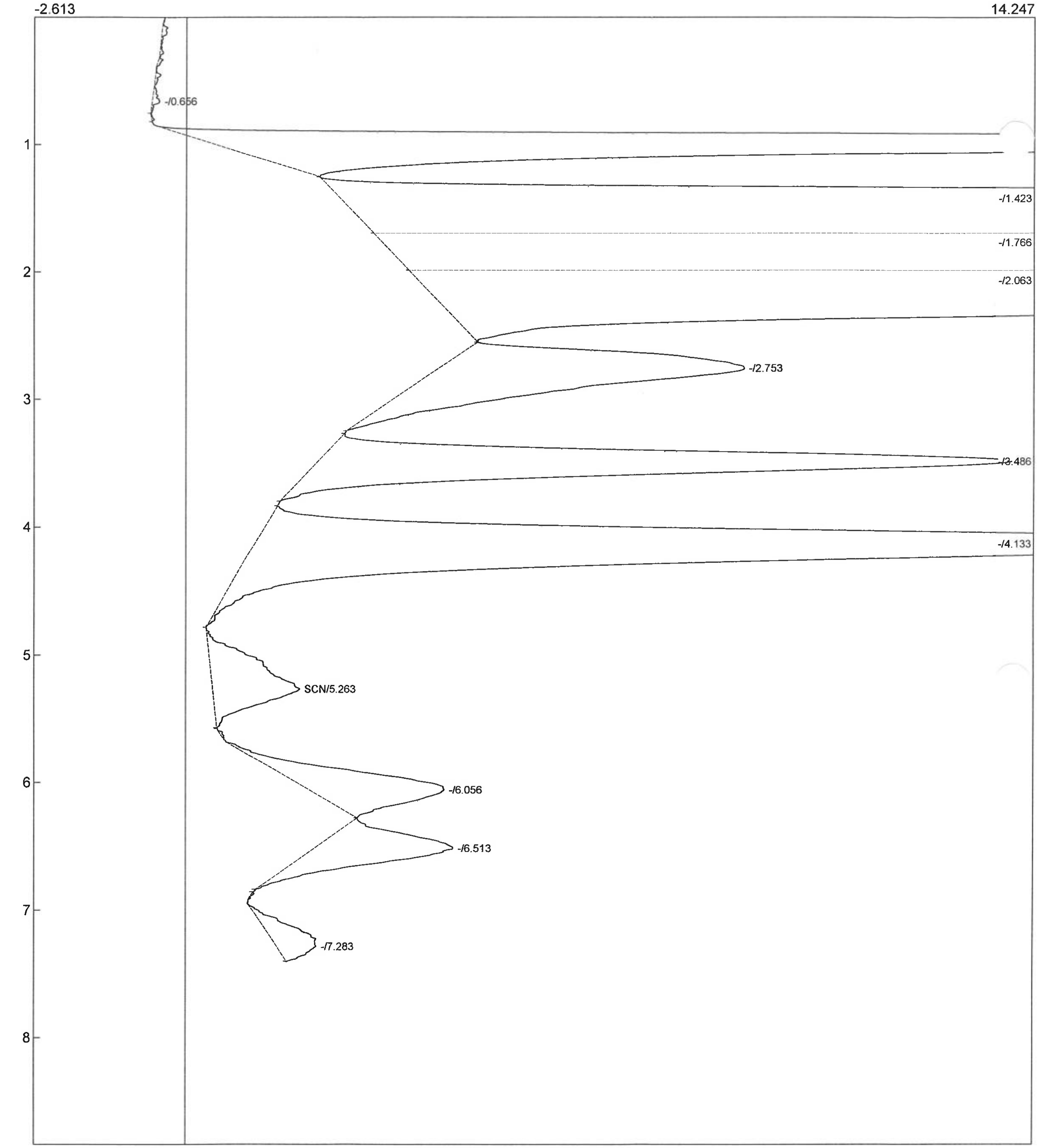

Component	Retention	Area	External	Units
SCN	5.263	31.3406	0.0000	
		31.3406	0.0000	

18-
4/23/15
PSI-12PC

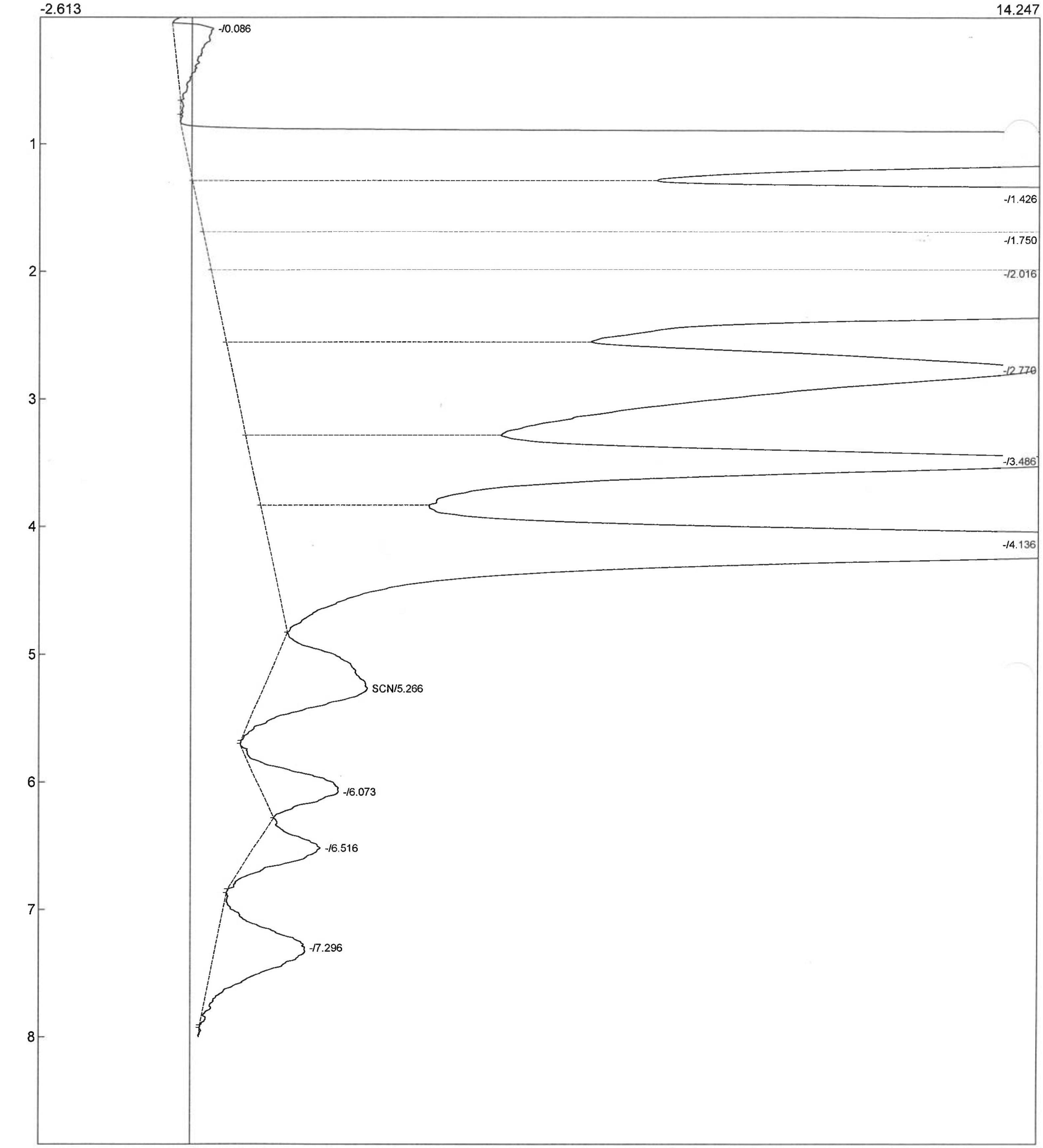

Component	Retention	Area	External	Units
SCN	5.266	44.6340	0.0000	
		44.6340	0.0000	

Lab name: RWU SCN Project
Client:
Client ID:
Analysis date: 08/02/2018 13:27:04
Method: Autosampler
Description: UV-Detector 220nm
Column: C30-UG-5 02021868-mx
Carrier: NaSO4 350mM 8/1
Integration: Peak sens=90.0 Base sens=40.0 Min area= 0.50 Standard= 1.000 Sample= 1.000 Tangents=off
Data file: SCN_Standard_Calibration_08022018_12.ASC ()
Sample:
Comments: TYPE YOUR COMMENTS HERE

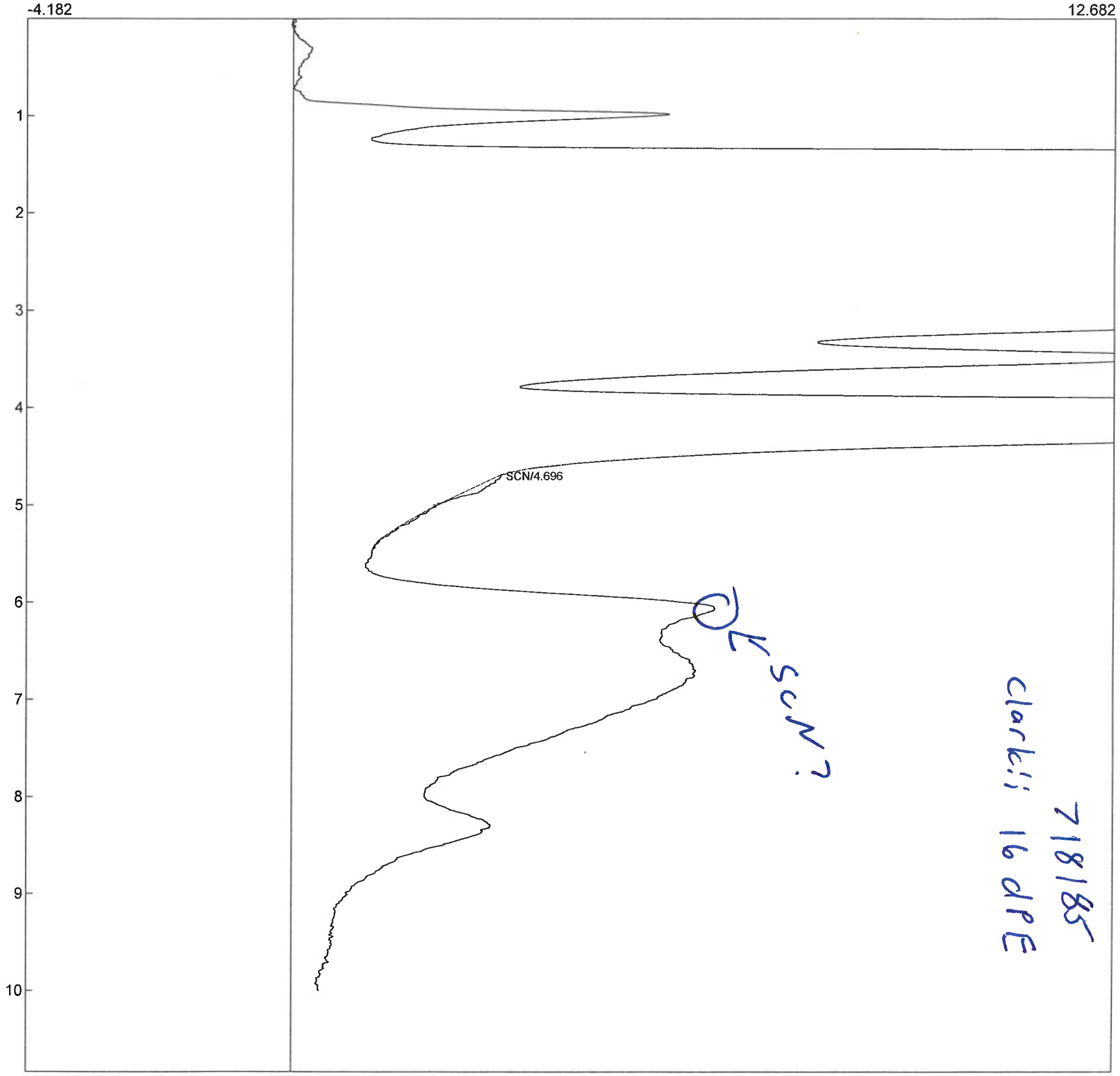

Component	Retention	Area	External	Units
SCN	4.696	2.2162	0.0000	
		2.2162	0.0000	

Lab name: RWU SCN Project
Client:
Client ID:
Analysis date: 08/02/2018 13:38:18
Method: Autosampler
Description: UV-Detector 220nm
Column: C30-UG-5 02021868-mx
Carrier: NaSO4 350mM 8/1
Integration: Peak sens=90.0 Base sens=40.0 Min area= 0.50 Standard= 1.000 Sample= 1.000 Tangents=off
Data file: SCN_Standard_Calibration_08022018_13.ASC ()
Sample:
Comments: TYPE YOUR COMMENTS HERE

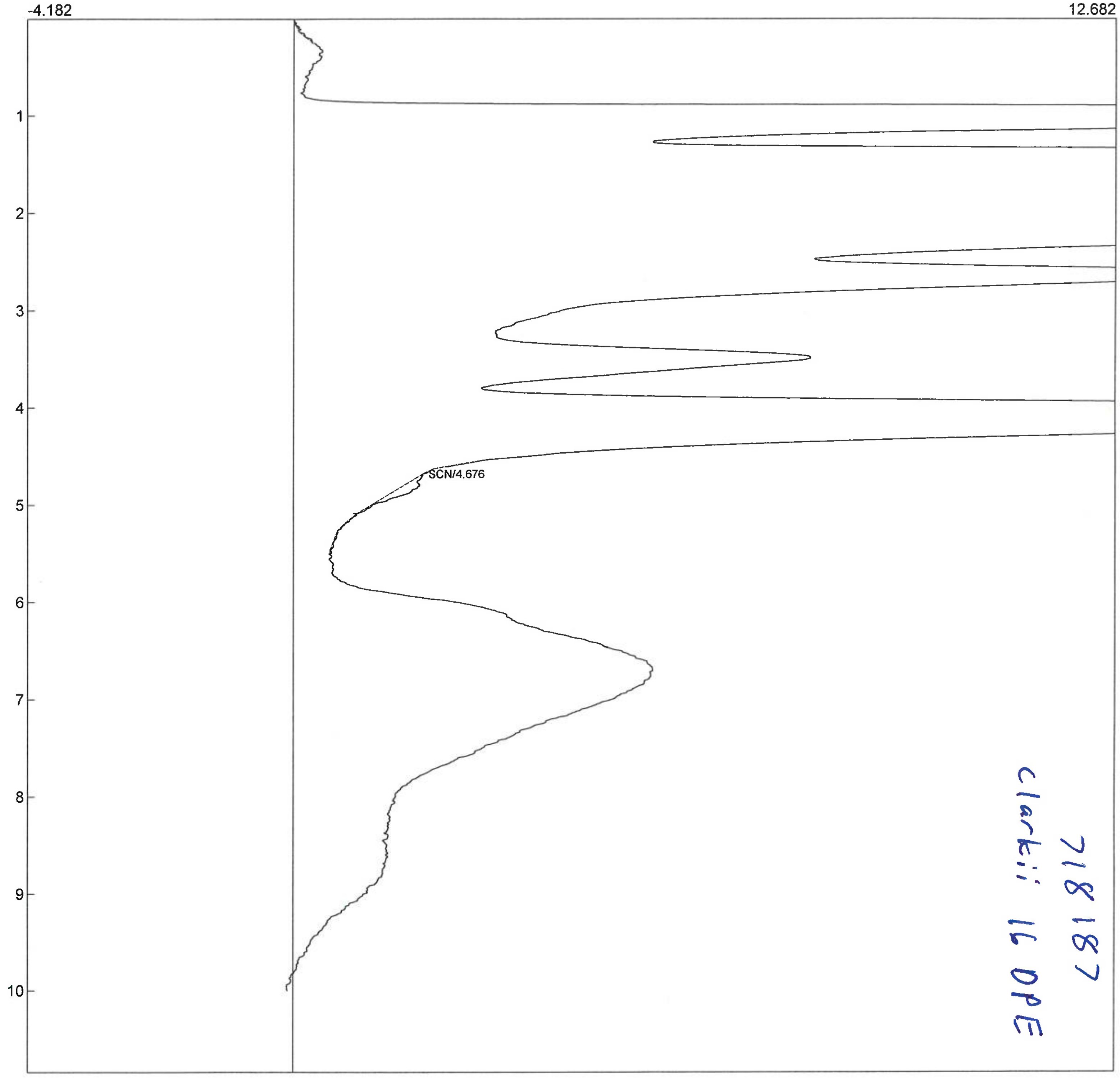

Component	Retention	Area	External	Units
SCN	4.676	3.2950	0.0000	
		3.2950	0.0000	

Lab name: RWU SCN Project
Client:
Client ID:
Analysis date: 08/02/2018 15:54:13
Method: Autosampler
Description: UV-Detector 220nm
Column: C30-UG-5 02021868-mx
Carrier: NaSO4 350mM 8/1
Integration: Peak sens=90.0 Base sens=40.0 Min area= 0.50 Standard= 1.000 Sample= 1.000 Tangents=off
Data file: SCN_Standard_Calibration_08022018_20.ASC ()
Sample:
Comments: TYPE YOUR COMMENTS HERE

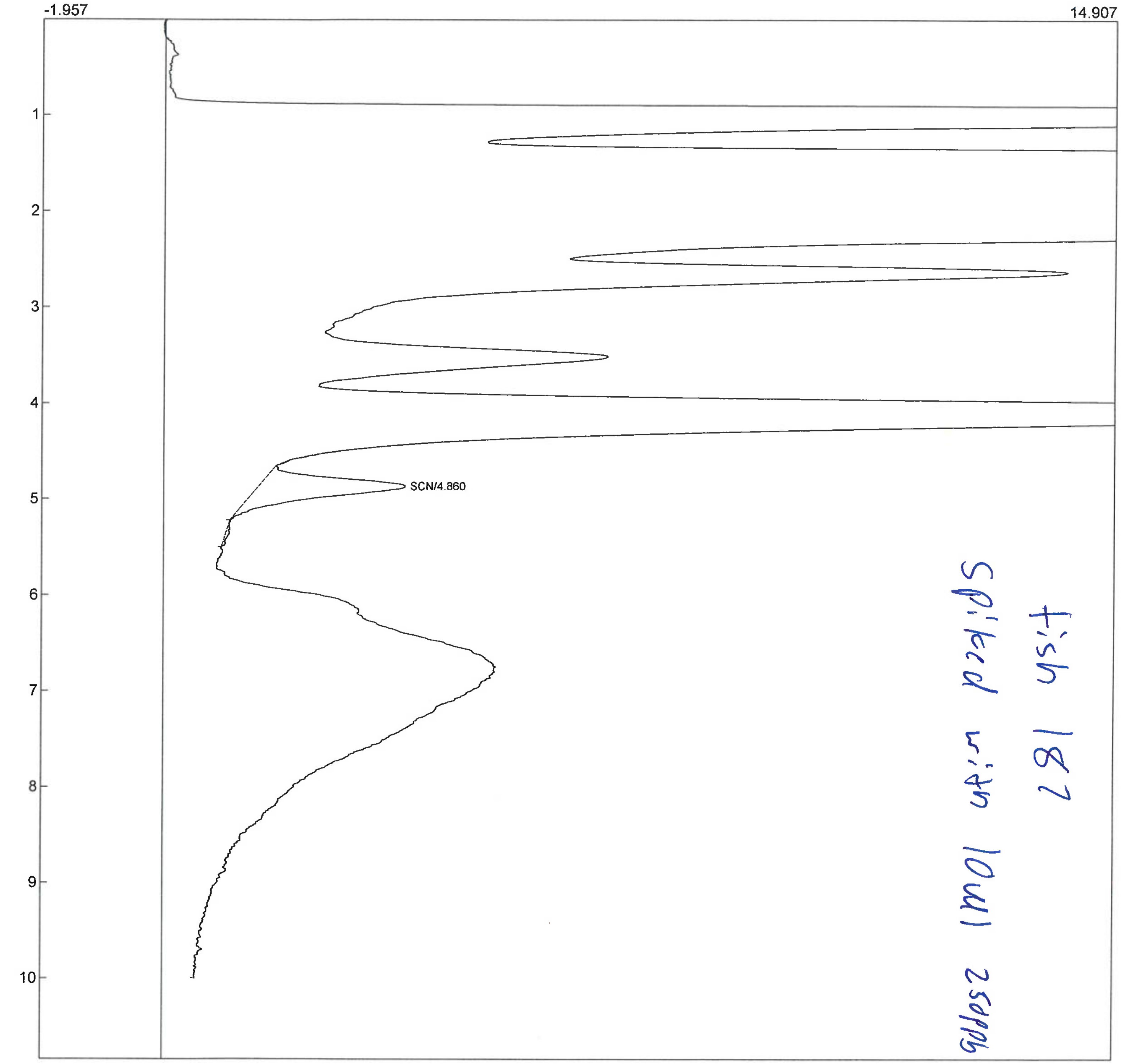

Component	Retention	Area	External	Units
SCN	4.860	30.4052	0.0000	
		30.4052	0.0000	

Lab name: RWU SCN Project
Client:
Client ID:
Analysis date: 08/03/2018 15:47:17
Method: Autosampler
Description: UV-Detector 220nm
Column: C30-UG-5 02021868-mx
Carrier: NaSO4 350mM 8/1
Integration: Peak sens=90.0 Base sens=40.0 Min area= 0.50 Standard= 1.000 Sample= 1.000 Tangents=off
Data file: SCN_Fish_08032018_11.ASC ()
Sample:
Comments: TYPE YOUR COMMENTS HERE

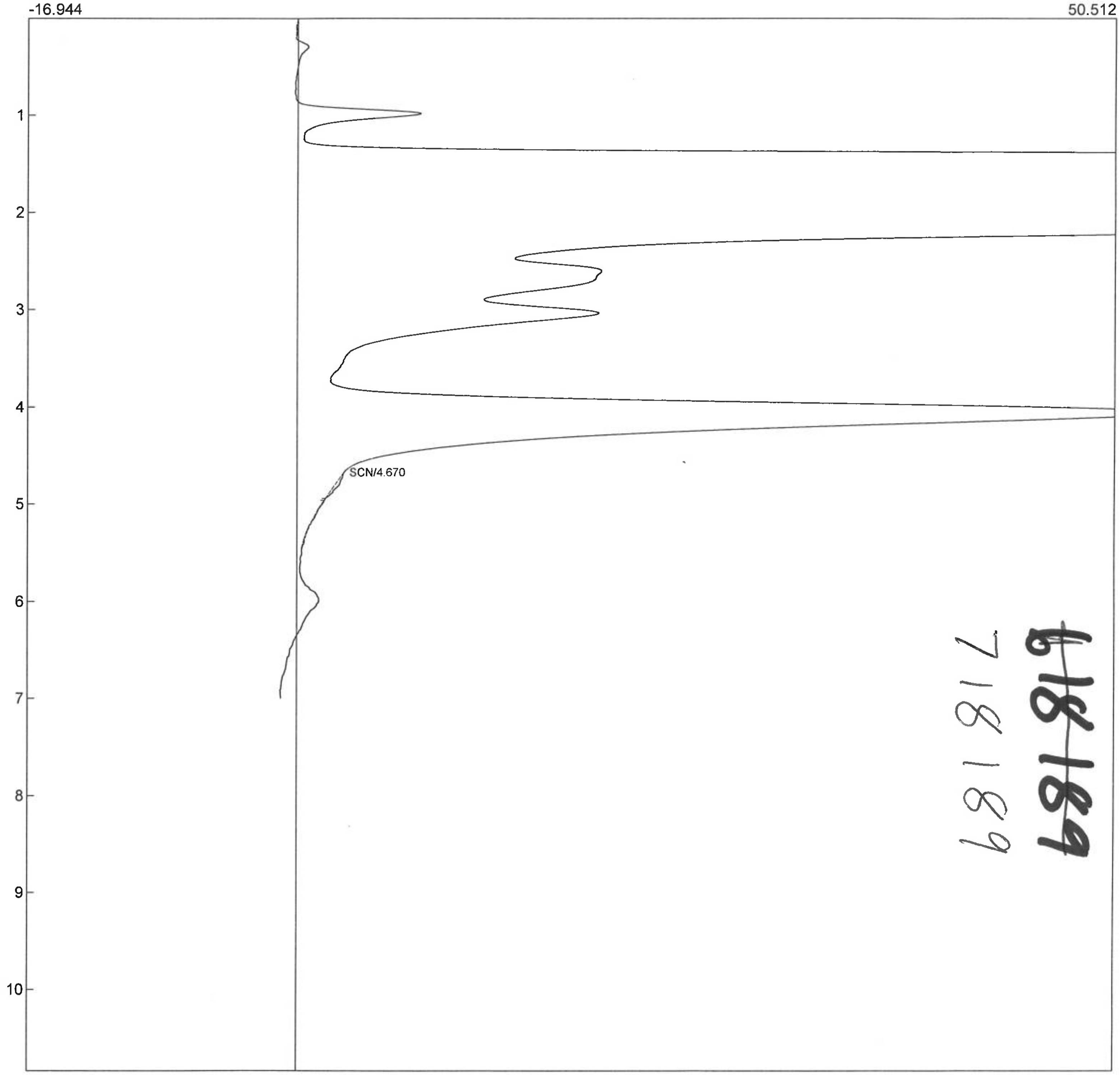

Component	Retention	Area	External	Units
SCN	4.670	2.5286	3.7476	
		2.5286	3.7476	

Lab name: RWU SCN Project
Client:
Client ID:
Analysis date: 08/03/2018 15:55:21
Method: Autosampler
Description: UV-Detector 220nm
Column: C30-UG-5 02021868-mx
Carrier: NaSO4 350mM 8/1
Integration: Peak sens=90.0 Base sens=40.0 Min area= 0.50 Standard= 1.000 Sample= 1.000 Tangents=off
Data file: SCN_Fish_08032018_12.ASC ()
Sample:
Comments: TYPE YOUR COMMENTS HERE

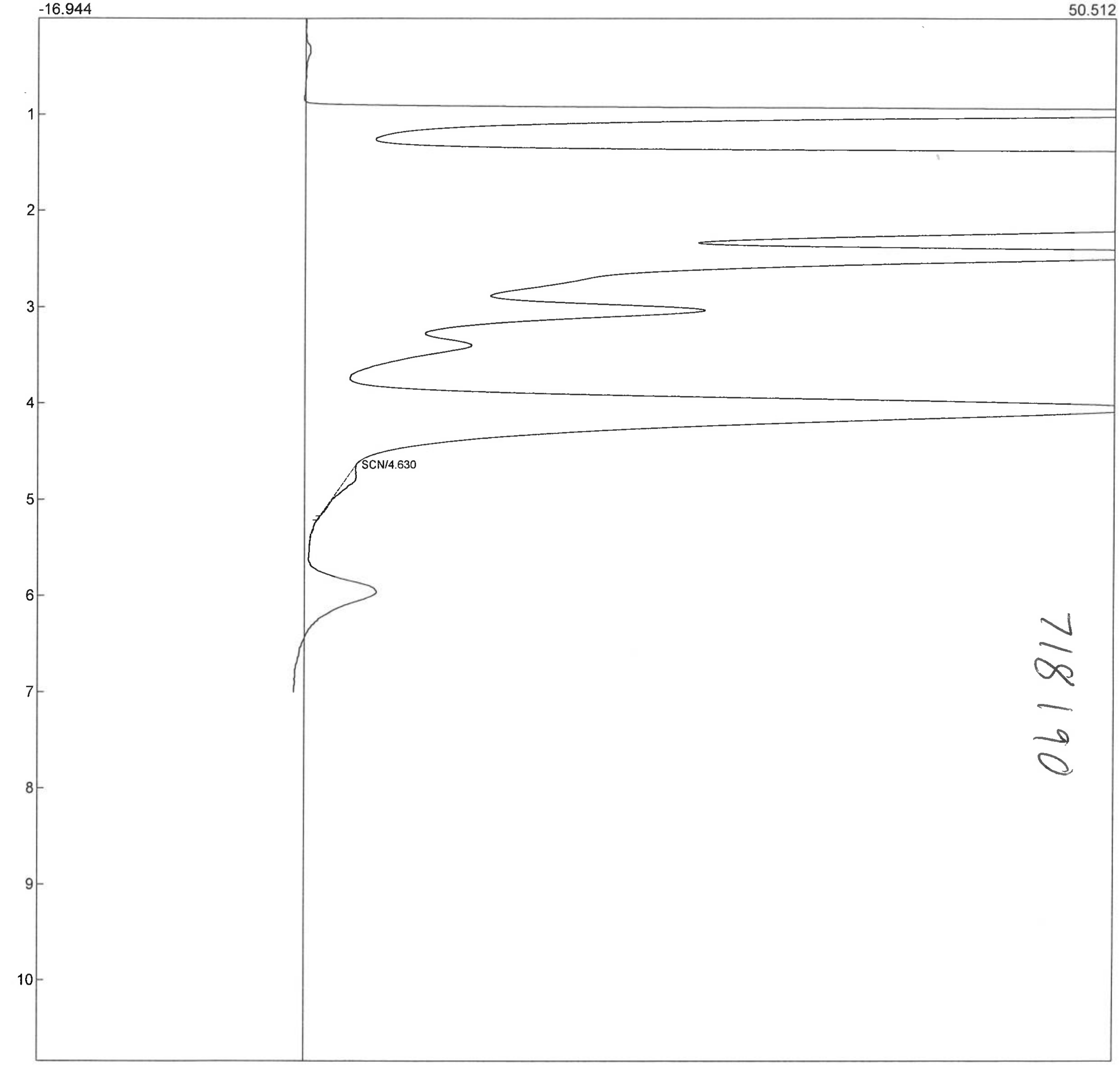

Component	Retention	Area	External	Units
SCN	4.630	8.1430	12.0685	
		8.1430	12.0685	

Lab name: RWU SCN Project
Client:
Client ID:
Analysis date: 08/03/2018 16:03:27
Method: Autosampler
Description: UV-Detector 220nm
Column: C30-UG-5 02021868-mx
Carrier: NaSO4 350mM 8/1
Integration: Peak sens=90.0 Base sens=40.0 Min area= 0.50 Standard= 1.000 Sample= 1.000 Tangents=off
Data file: SCN_Fish_08032018_13.ASC ()
Sample:
Comments: TYPE YOUR COMMENTS HERE

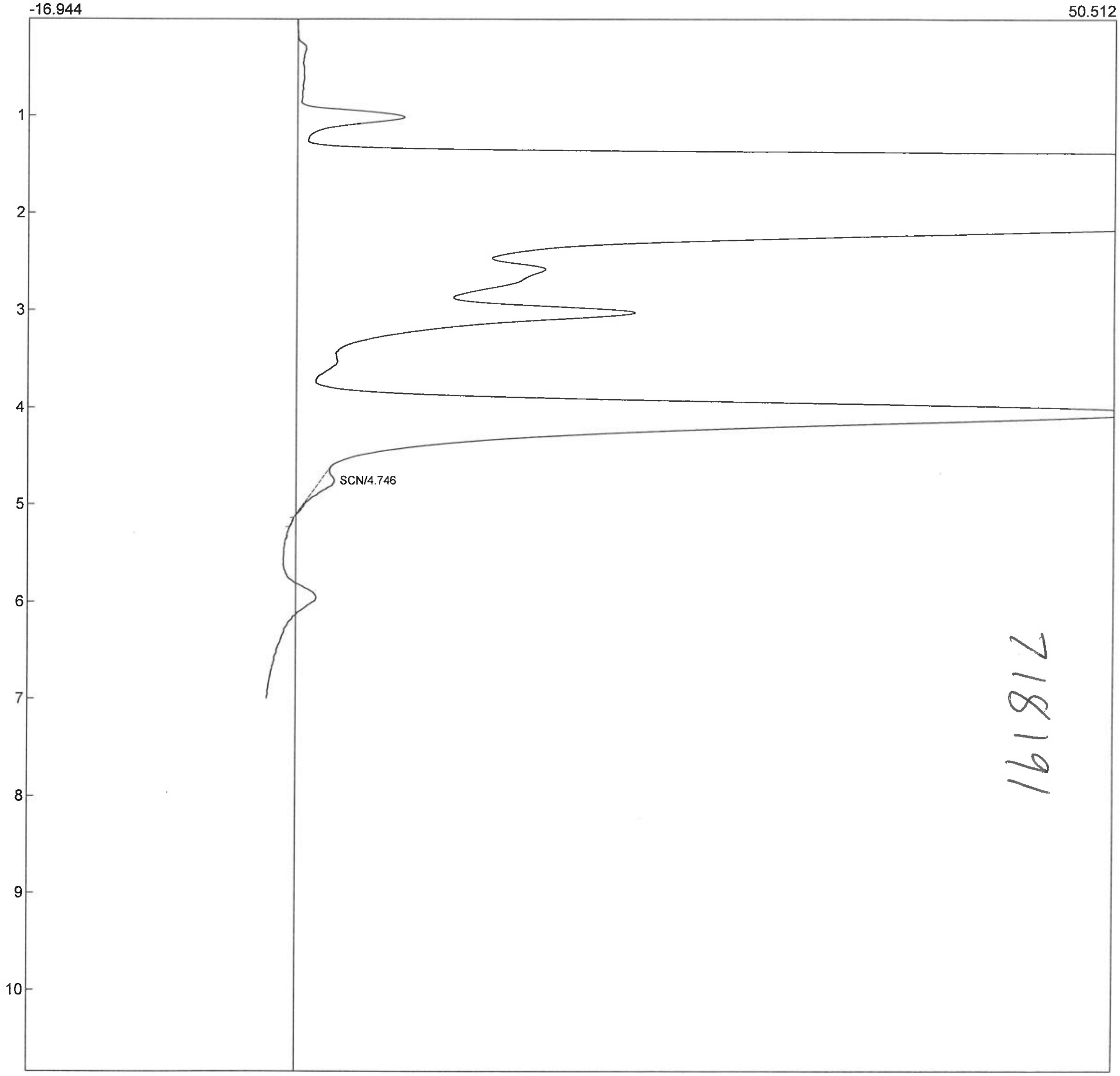

Component	Retention	Area	External	Units
SCN	4.746	11.0671	16.4022	
		11.0671	16.4022	

Lab name: RWU SCN Project
Client:
Client ID:
Analysis date: 08/03/2018 16:11:32
Method: Autosampler
Description: UV-Detector 220nm
Column: C30-UG-5 02021868-mx
Carrier: NaSO4 350mM 8/1
Integration: Peak sens=90.0 Base sens=40.0 Min area= 0.50 Standard= 1.000 Sample= 1.000 Tangents=off
Data file: SCN_Fish_08032018_14.ASC ()
Sample:
Comments: TYPE YOUR COMMENTS HERE

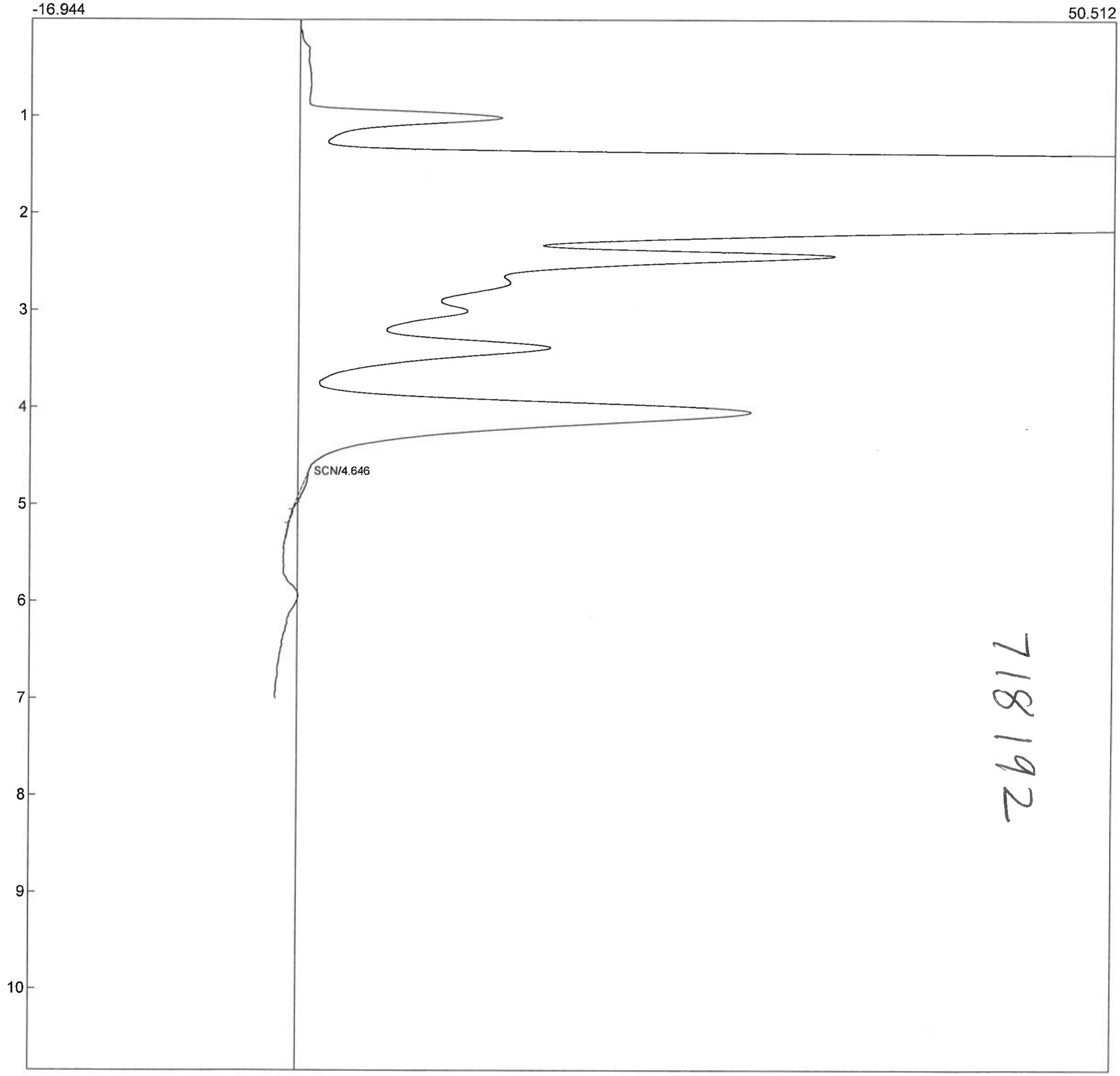

Component	Retention	Area	External	Units
SCN	4.646	3.2982	4.8882	
		3.2982	4.8882	

Lab name: RWU SCN Project
Client:
Client ID:
Analysis date: 08/03/2018 16:19:43
Method: Autosampler
Description: UV-Detector 220nm
Column: C30-UG-5 02021868-mx
Carrier: NaSO4 350mM 8/1
Integration: Peak sens=90.0 Base sens=40.0 Min area= 0.50 Standard= 1.000 Sample= 1.000 Tangents=off
Data file: SCN_Fish_08032018_15.ASC ()
Sample:
Comments: TYPE YOUR COMMENTS HERE

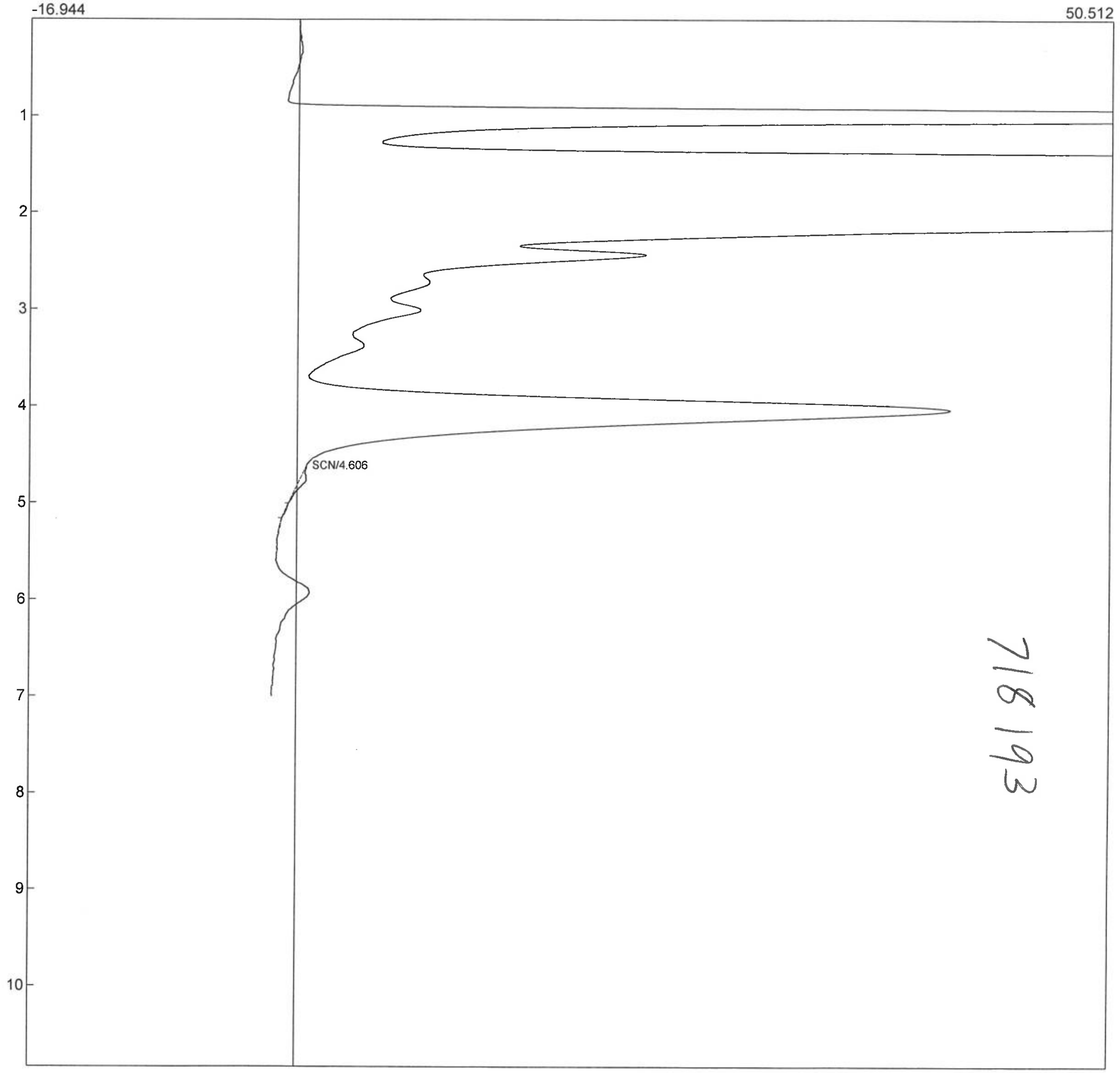

Component	Retention	Area	External	Units
SCN	4.606	4.2341	6.2752	
		4.2341	6.2752	

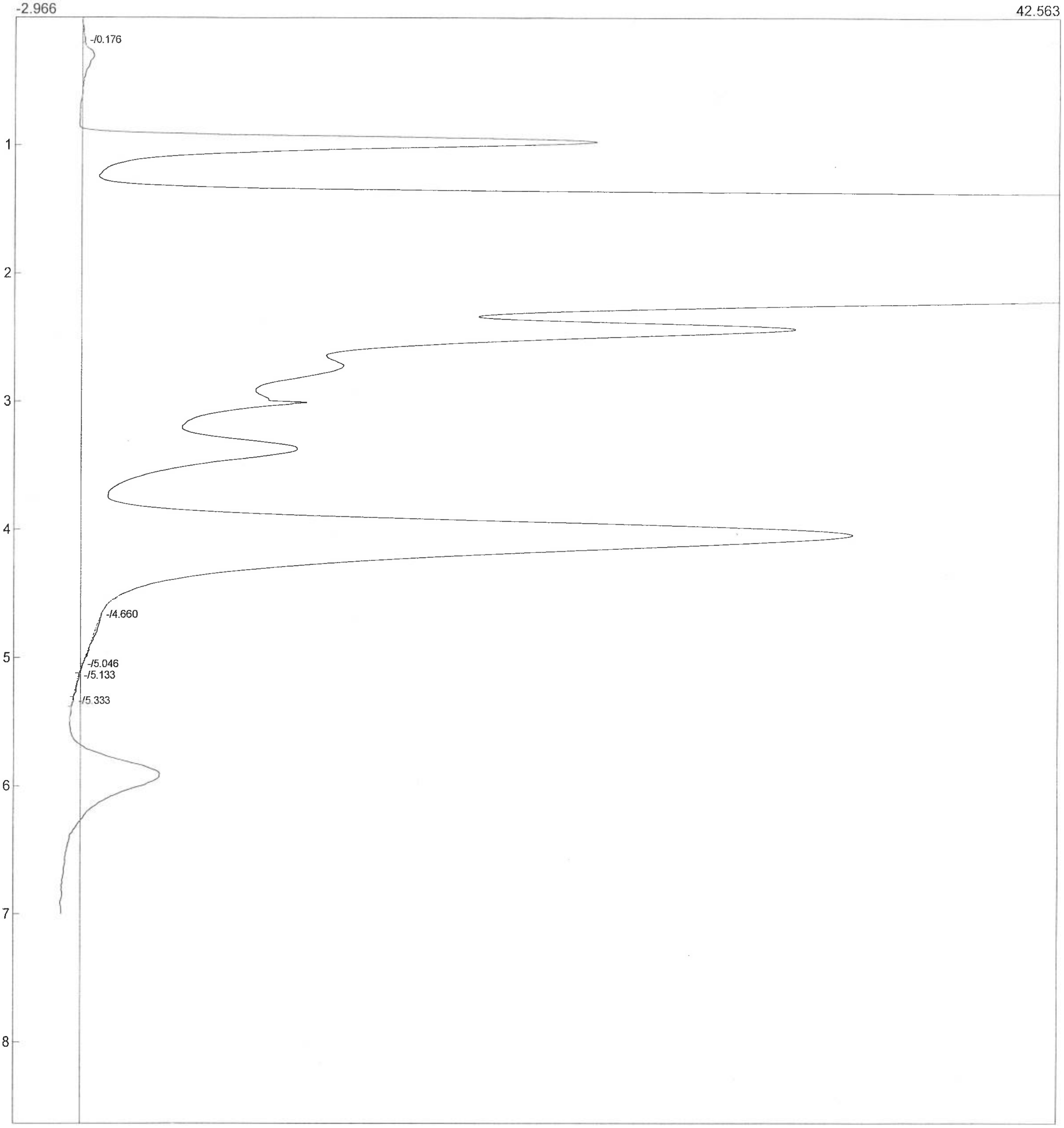

Reprinted
7/8/94

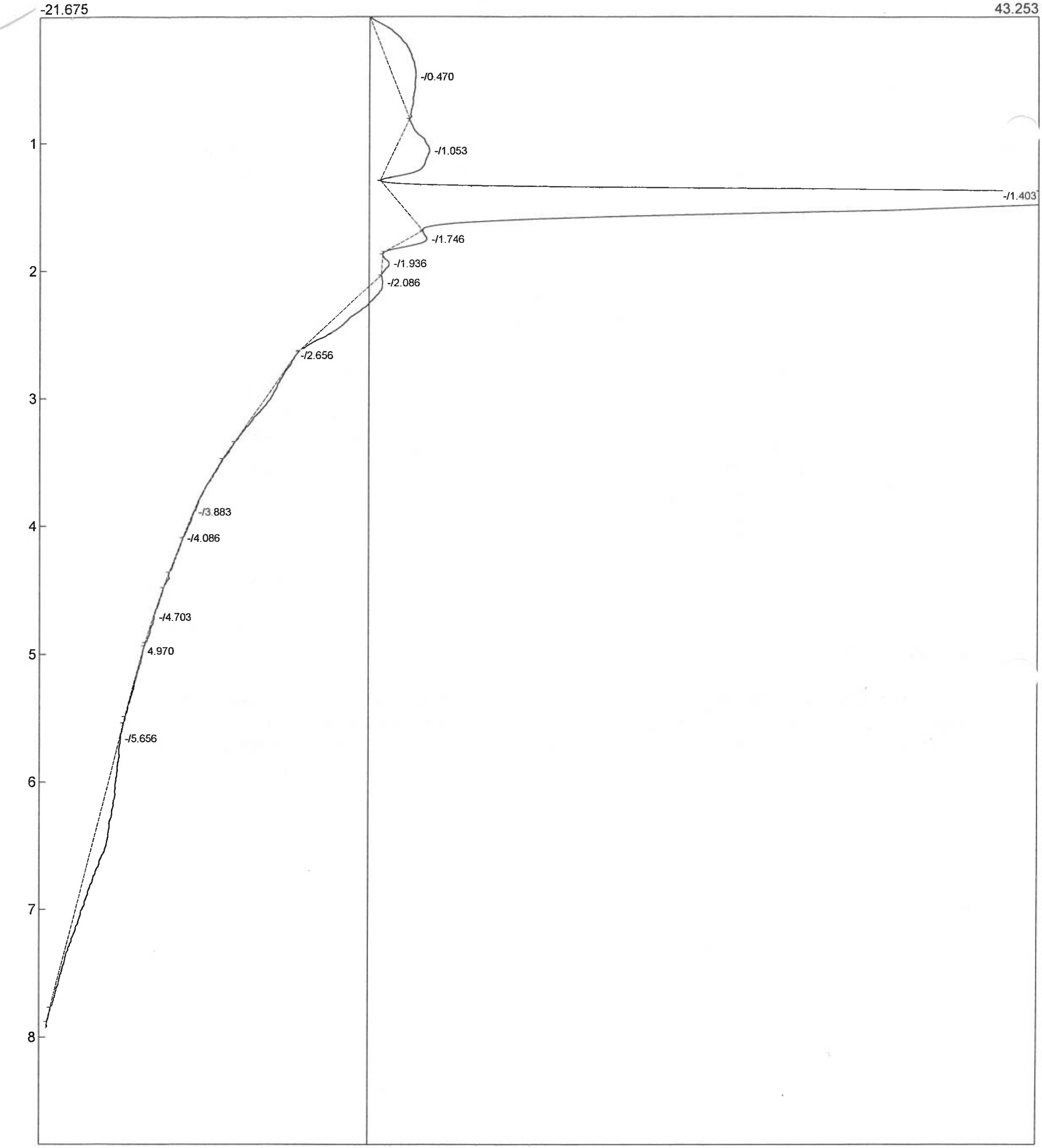

Component	Retention	Area	External	Units
	4.970	1.5012	0.0000	
		1.5012	0.0000	

blank
2/15/18
PSI-1513

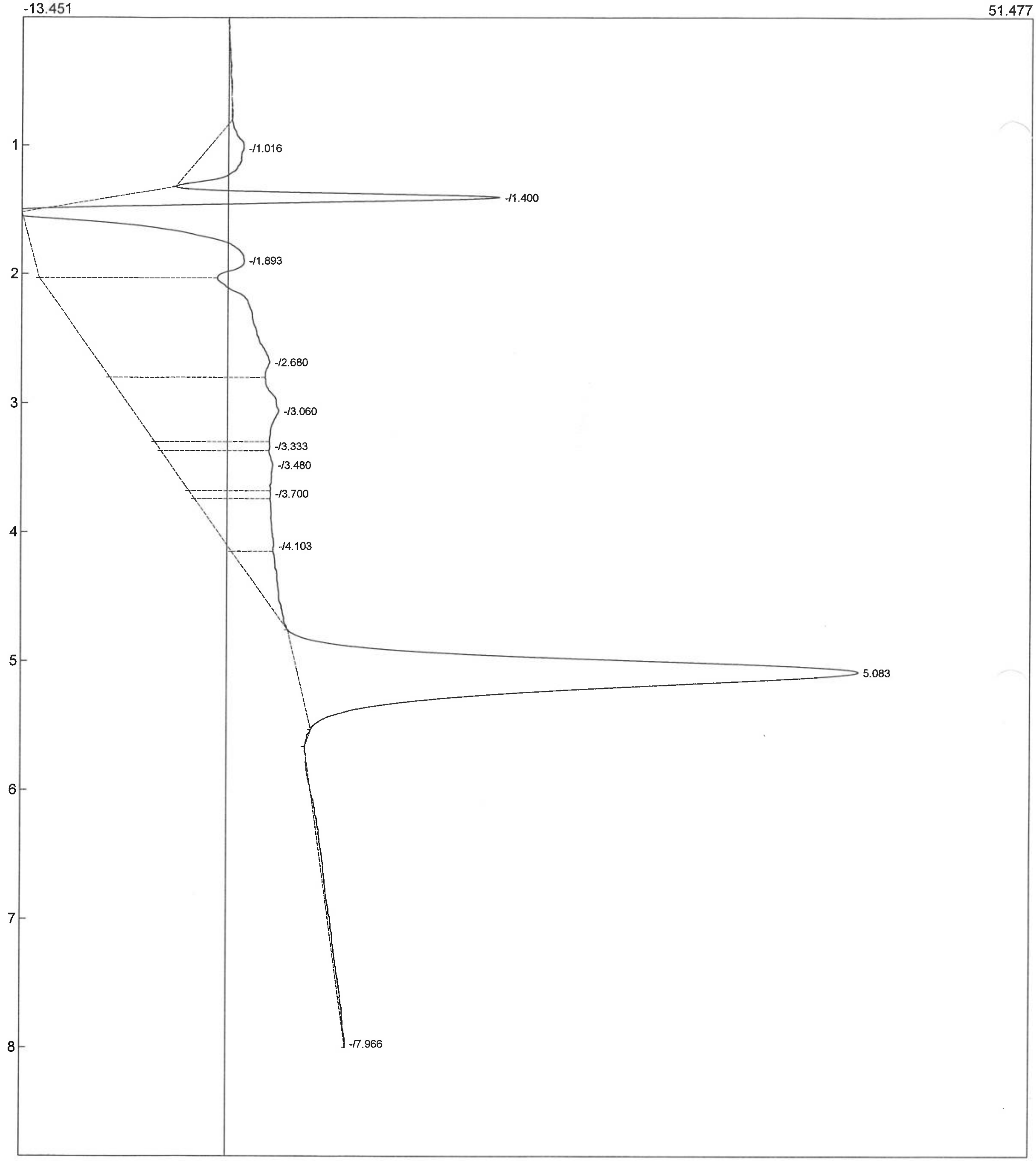

Component	Retention	Area	External	Units
	5.083	569.5966	0.0000	
		569.5966	0.0000	

7/18/16
11pm

Lab name: RWU SCN Project
Client:
Client ID:
Analysis date: 08/06/2018 12:42:11
Method: Autosampler
Description: UV-Detector 220nm
Column: C30-UG-5 02021868-mx
Carrier: NaSO4 350mM 8/1
Integration: Peak sens=90.0 Base sens=40.0 Min area= 0.50 Standard= 1.000 Sample= 1.000 Tangents=off
Data file: SCN_Calibration_08062018_07.ASC ()
Sample:
Comments: TYPE YOUR COMMENTS HERE

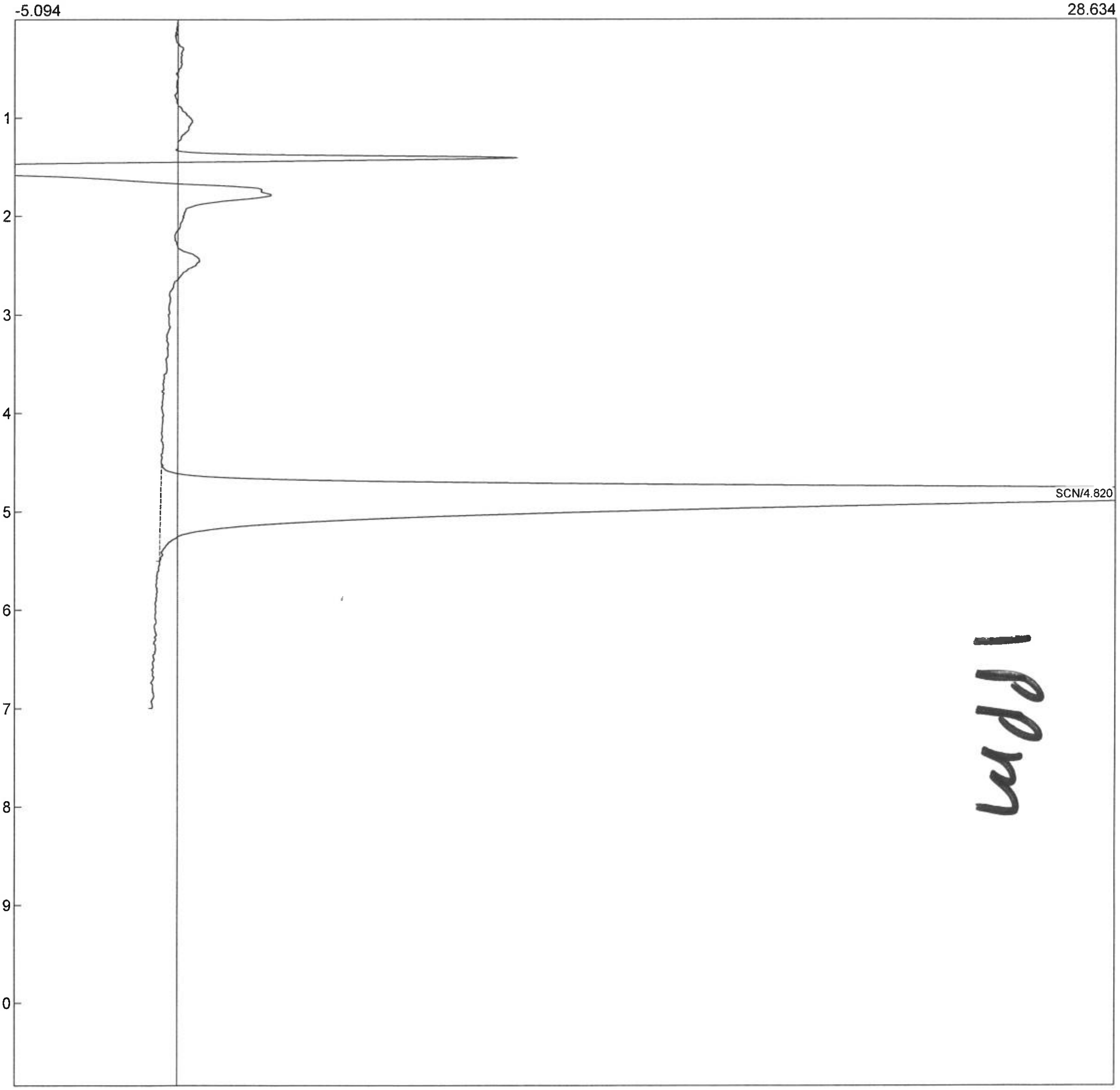

Component	Retention	Area	External	Units
SCN	4.820	587.4732	870.6781	
		587.4732	870.6781	

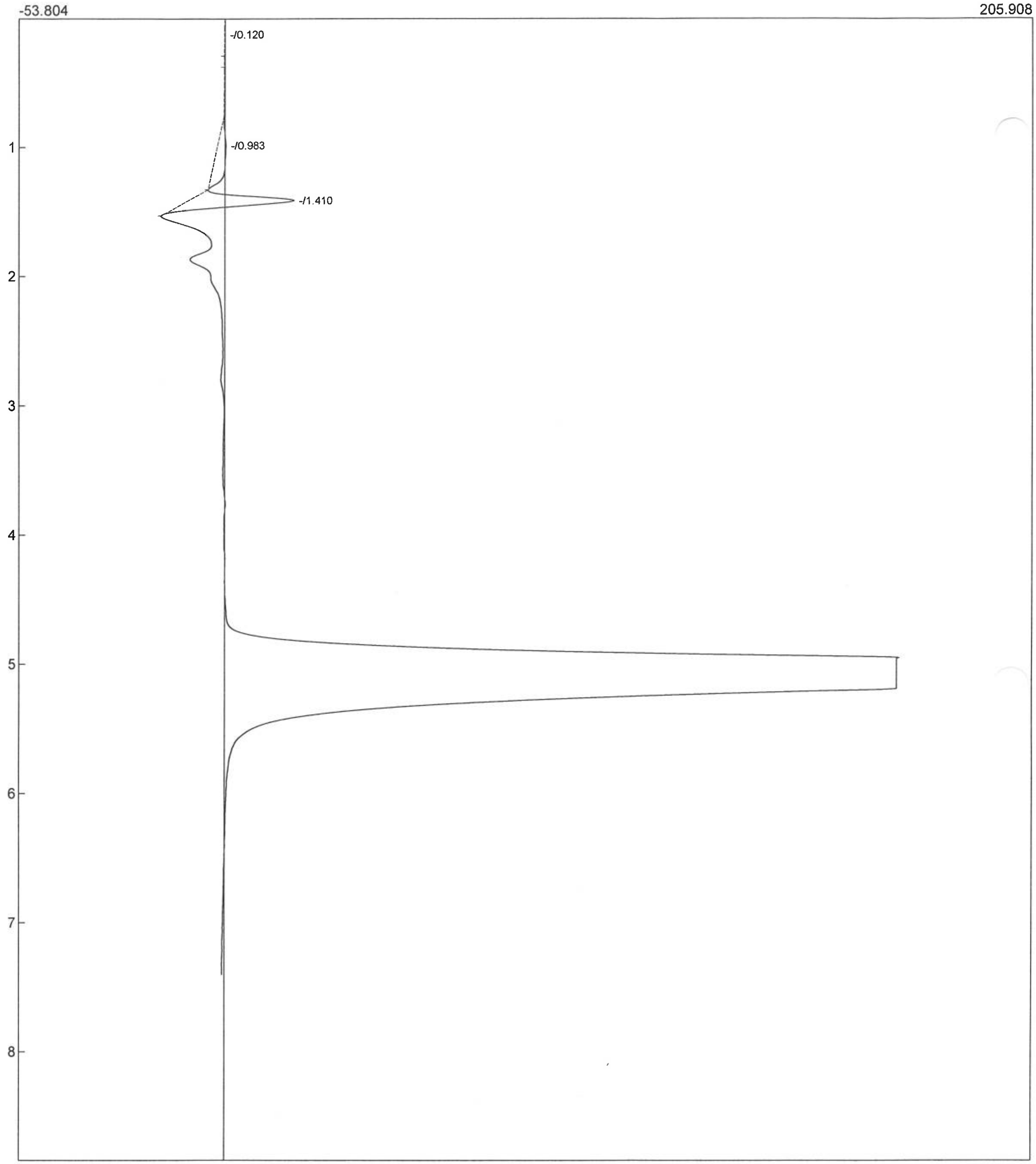

Component	Retention	Area	External	Units
	0.000	0.0000	0.0000	
		0.0000	0.0000	

7/18/18

10 ppm

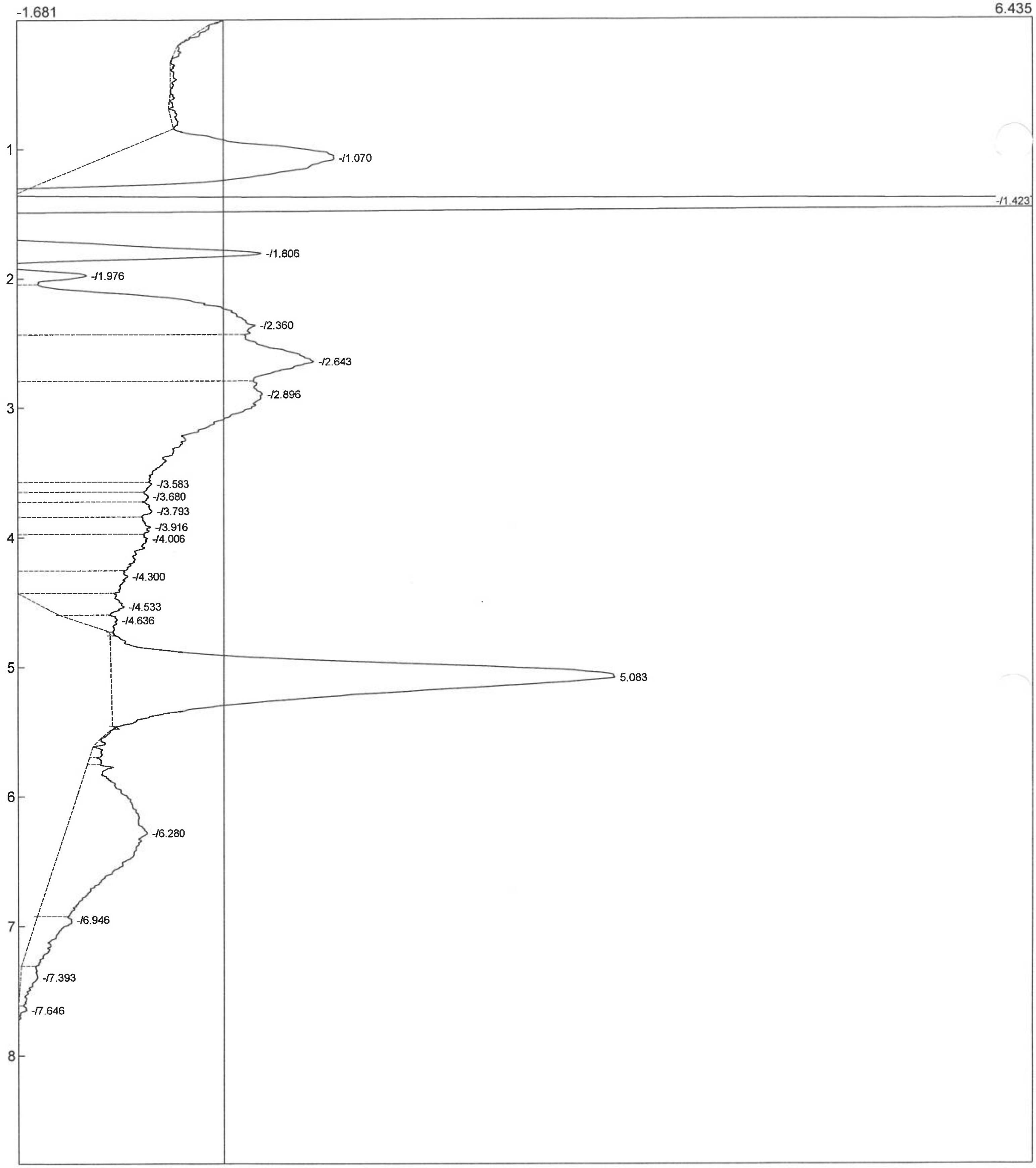

Component	Retention	Area	External	Units
	5.083	66.9300	0.0000	
		66.9300	0.0000	

7/18/18
100ppb

Lab name: RWU SCN Project
Client:
Client ID:
Analysis date: 08/02/2018 13:49:30
Method: Autosampler
Description: UV-Detector 220nm
Column: C30-UG-5 02021868-mx
Carrier: NaSO4 350mM 8/1
Integration: Peak sens=90.0 Base sens=40.0 Min area= 0.50 Standard= 1.000 Sample= 1.000 Tangents=off
Data file: SCN_Standard_Calibration_08022018_14.ASC ()
Sample:
Comments: TYPE YOUR COMMENTS HERE

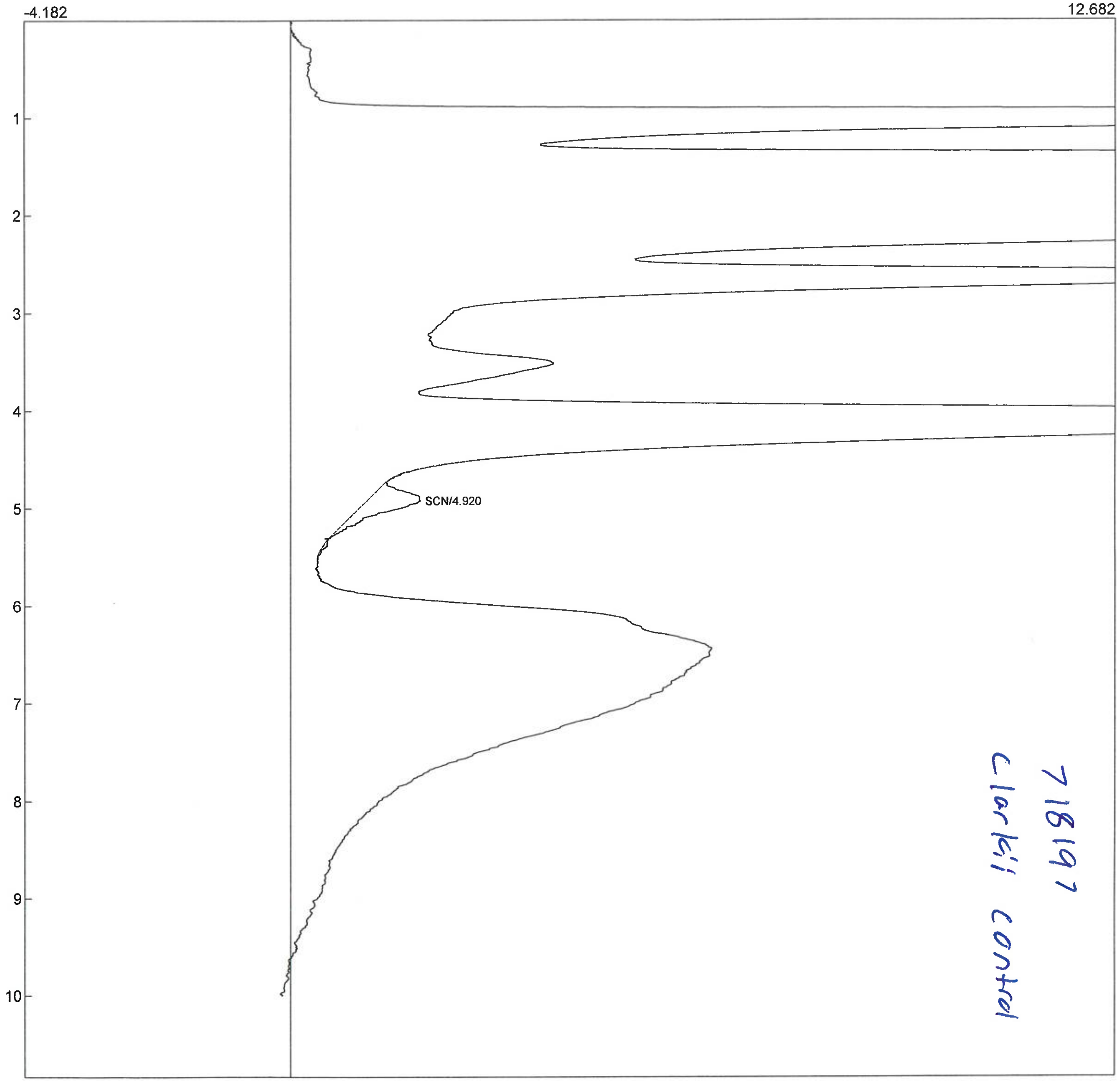

Component	Retention	Area	External	Units
SCN	4.920	12.4699	0.0000	
		12.4699	0.0000	

Lab name: RWU SCN Project
Client:
Client ID:
Analysis date: 08/02/2018 14:00:43
Method: Autosampler
Description: UV-Detector 220nm
Column: C30-UG-5 02021868-mx
Carrier: NaSO4 350mM 8/1
Integration: Peak sens=90.0 Base sens=40.0 Min area= 0.50 Standard= 1.000 Sample= 1.000 Tangents=off
Data file: SCN_Standard_Calibration_08022018_15.ASC ()
Sample:
Comments: TYPE YOUR COMMENTS HERE

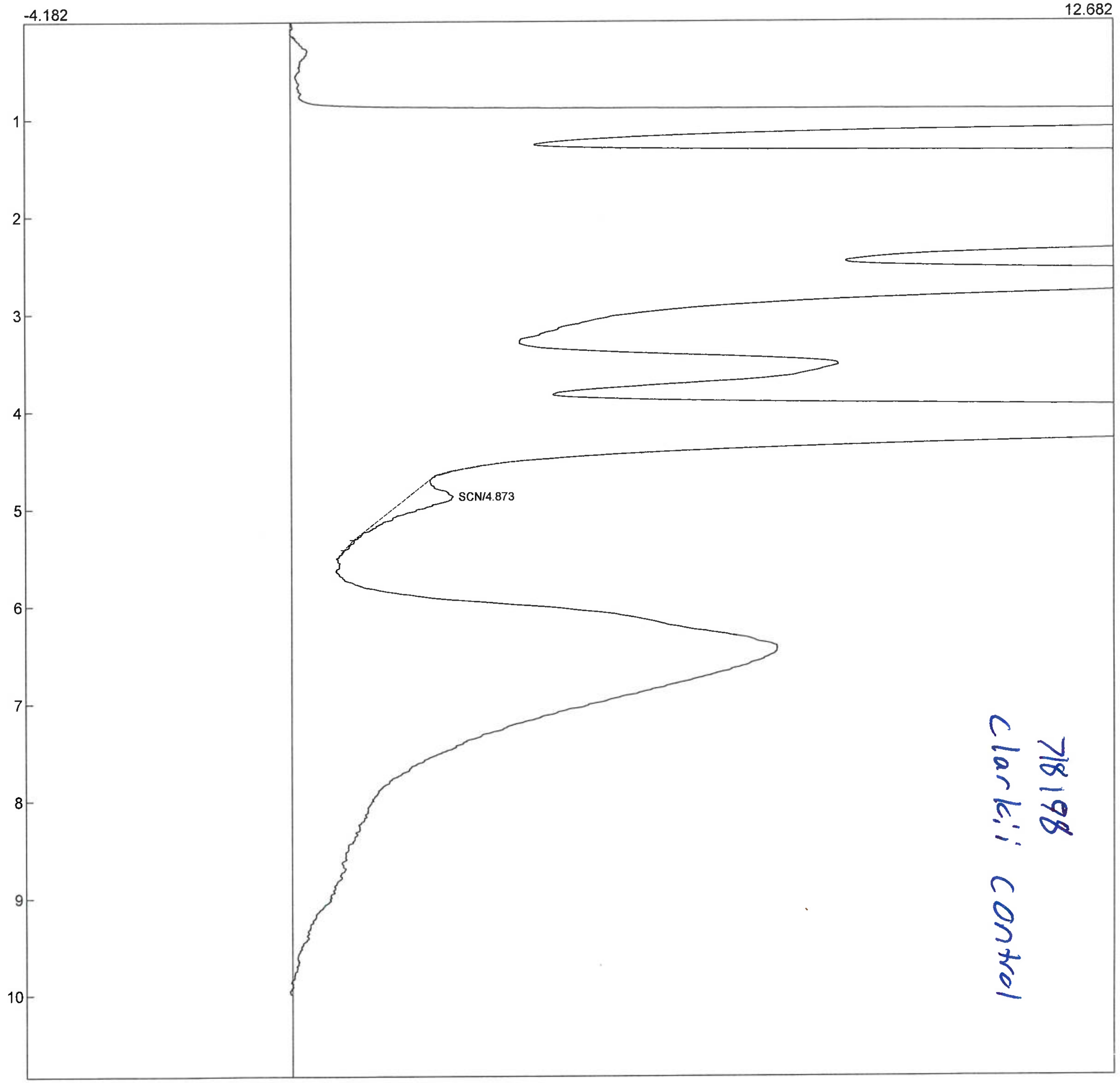

Component	Retention	Area	External	Units
SCN	4.873	10.7934	0.0000	
		10.7934	0.0000	

Lab name: RWU SCN Project
Client:
Client ID:
Analysis date: 08/02/2018 14:11:57
Method: Autosampler
Description: UV-Detector 220nm
Column: C30-UG-5 02021868-mx
Carrier: NaSO4 350mM 8/1
Integration: Peak sens=90.0 Base sens=40.0 Min area= 0.50 Standard= 1.000 Sample= 1.000 Tangents=off
Data file: SCN_Standard_Calibration_08022018_16.ASC ()
Sample:
Comments: TYPE YOUR COMMENTS HERE

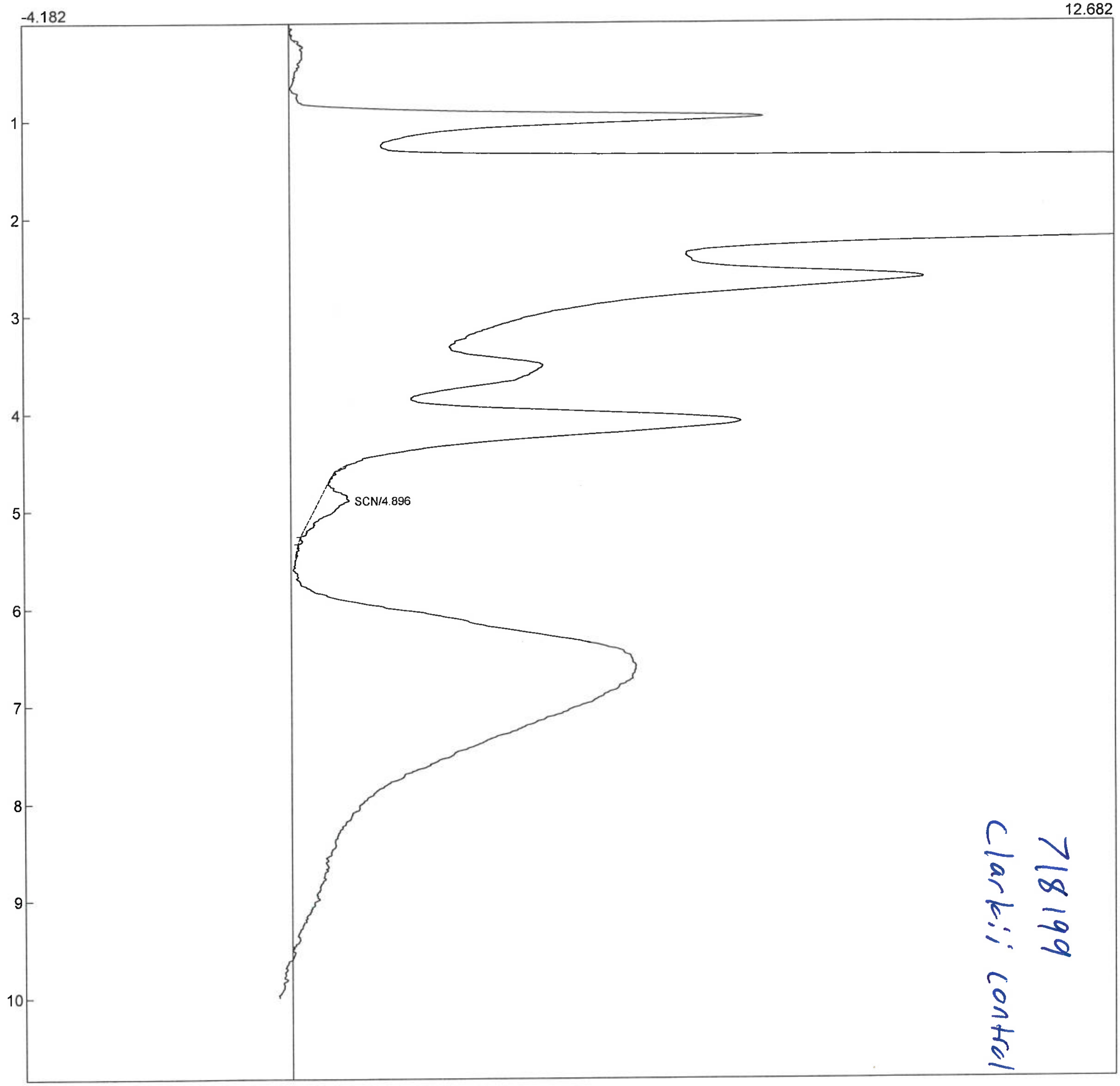

Component	Retention	Area	External	Units
SCN	4.896	7.1888	0.0000	
		7.1888	0.0000	

Lab name: RWU SCN Project
Client:
Client ID:
Analysis date: 08/02/2018 14:23:10
Method: Autosampler
Description: UV-Detector 220nm
Column: C30-UG-5 02021868-mx
Carrier: NaSO4 350mM 8/1
Integration: Peak sens=90.0 Base sens=40.0 Min area= 0.50 Standard= 1.000 Sample= 1.000 Tangents=off
Data file: SCN_Standard_Calibration_08022018_17.ASC ()
Sample:
Comments: TYPE YOUR COMMENTS HERE

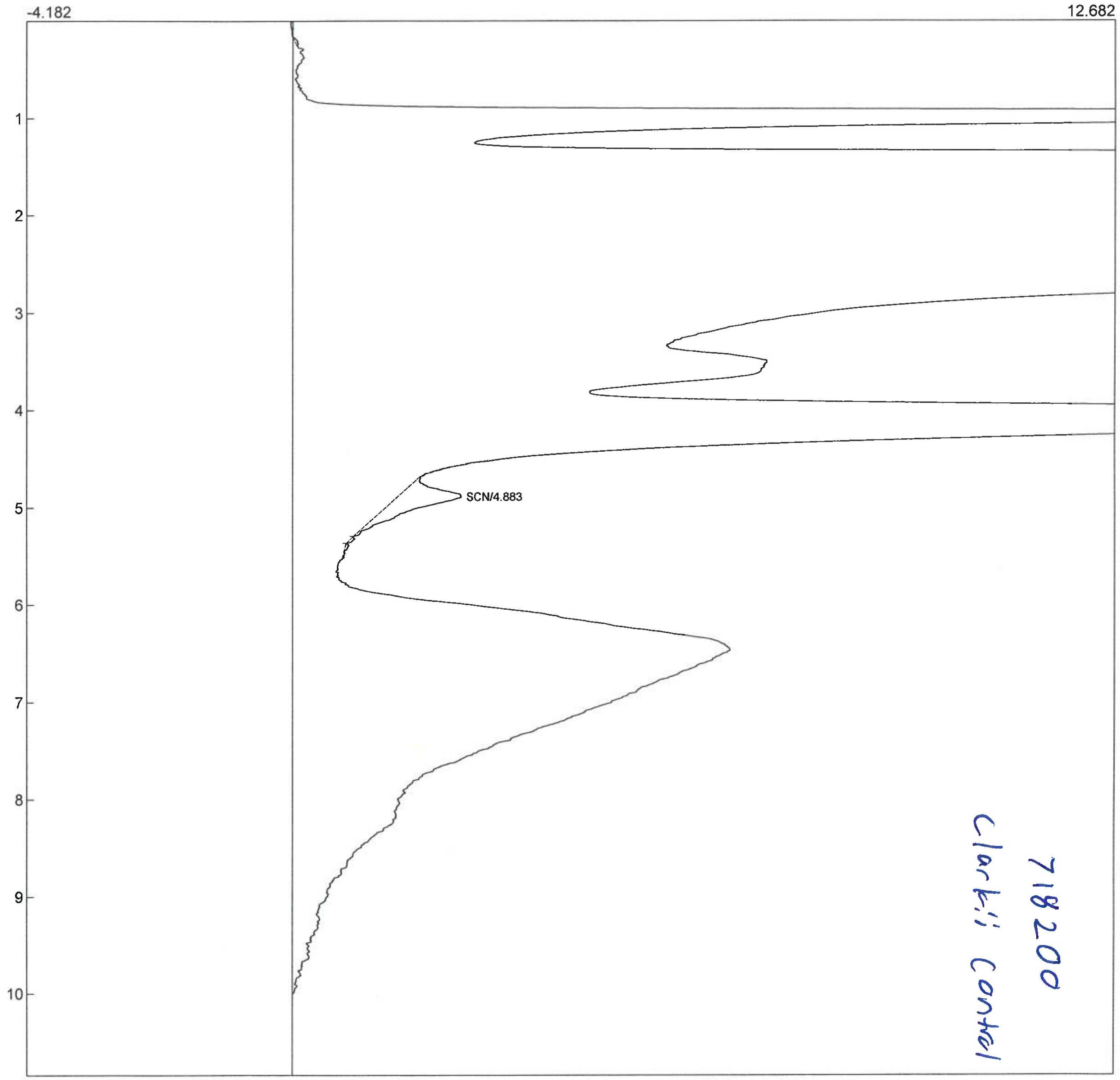

Component	Retention	Area	External	Units
SCN	4.883	14.2421	0.0000	
		14.2421	0.0000	
